# Integrated Transcriptome Analysis Reveals Novel Molecular Signatures for Schizophrenia Characterization

**DOI:** 10.1002/advs.202407628

**Published:** 2024-11-20

**Authors:** Tong Ni, Yu Sun, Zefeng Li, Tao Tan, Wei Han, Miao Li, Li Zhu, Jing Xiao, Huiying Wang, Wenpei Zhang, Yitian Ma, Biao Wang, Di Wen, Teng Chen, Justin Tubbs, Xiaofeng Zeng, Jiangwei Yan, Hongsheng Gui, Pak Sham, Fanglin Guan

**Affiliations:** ^1^ Key Laboratory of National Health Commission for Forensic Sciences Xi'an Jiaotong University Health Science Center Xi'an 710061 China; ^2^ Institute of Neuroscience Bio‐evidence Sciences Academy Xi'an Jiaotong University Health Science Center Xi'an 712046 China; ^3^ Department of Endocrinology and Metabolism Qilu Hospital of Shandong University Ji'nan 250000 China; ^4^ Oujiang Laboratory (Zhejiang Lab for Regenerative Medicine Vision and Brain Health) Key Laboratory of Alzheimer's Disease of Zhejiang Province Institute of Aging Wenzhou Medical University Wenzhou 325603 China; ^5^ Department of Ultrasound the Second Affiliated Hospital Xi'an Jiaotong University Xi'an 710004 China; ^6^ Department of Immunology and Pathogenic Biology College of Basic Medicine Xi'an Jiaotong University Health Science Center Xi'an 710061 China; ^7^ College of Forensic Medicine Hebei Key Laboratory of Forensic Medicine Hebei Medical University Shijiazhuang 050017 China; ^8^ Department of Psychiatry Li Ka Shing Faculty of Medicine the University of Hong Kong Hong Kong SAR 999077 China; ^9^ Department of Forensic Medicine School of Forensic Medicine Kunming Medical University Kunming 650500 China; ^10^ Department of Genetics, School of Medicine & Forensics Shanxi Medical University Taiyuan 030009 China; ^11^ Behavioral Health Services and Psychiatry Research Henry Ford Health Detroit MI 48202 USA; ^12^ Department of Psychiatry Michigan State University East Lansing MI 48824 USA

**Keywords:** characterization, machine learning, molecular signatures, schizophrenia, transcriptome

## Abstract

Schizophrenia (SCZ) is a complex psychiatric disorder presenting challenges for characterization. The current study aimed to identify and evaluate disease‐responsive essential genes (DREGs) to enhance the molecular characterization of SCZ. RNA‐sequencing data from PsychENCODE (536 SCZ patients, 832 controls) and peripheral blood transcriptome data from 144 recruited subjects (59 SCZ patients, 6 non‐SCZ psychiatric patients, 79 controls) are analyzed. Shared differential expression genes are obtained using three algorithms. Support vector machine (SVM)‐based recursive feature elimination is employed to identify DREGs. The biological relevance of these DREGs is examined through protein–protein interaction network, pathway enrichment, polygenic scoring, and brain tissue expression. Key DREGs are validated in SCZ animal models. A DREGs‐based machine‐learning model for SCZ characterization is developed and its performance is assessed using multiple datasets. The analysis identified 184 DREGs forming an interconnected network involved in synaptic plasticity, inflammation, neuronal development, and neurotransmission. DREGs exhibited distinct expression in SCZ‐related brain regions and animal models. Their genetic contributions are comparable to genome‐wide polygenic risk scores. The DREG‐based SVM model demonstrated high performance (AUC 85% for SCZ characterization, 79% for specificity). These findings provide new insights into the molecular mechanisms underlying SCZ and emphasize the potential of DREGs in improving SCZ characterization.

## Graphical abstract

This study leverages machine learning‐based transcriptomic analysis integrated with genomic annotation and experimental validation to identify 184 disease‐responsive essential genes (DREGs) in schizophrenia. These DREGs form an interconnected network involved in synaptic plasticity, neuroinflammation, and neurodevelopment. The developed DREG‐based model demonstrates high performance in disease characterization, offering new insights into molecular mechanisms and potential therapeutic targets.

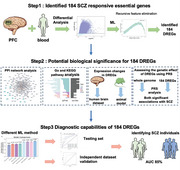

## 1 Introduction

Schizophrenia (SCZ) is a complex psychiatric disorder with a significant societal burden, affecting roughly 0.3% of the population and characterized by a combination of psychotic symptoms, cognitive deficits, and functional impairments.^[^ ^1^ ^]^ Understanding the underlying pathogenic mechanisms of SCZ is crucial for improving diagnosis, treatment, and patient outcomes. Considerable progress has been made in identifying genetic risk factors through genome‐wide association studies (GWAS).^[^ ^2^, ^3^ ^]^ These studies have provided valuable insights into the genetic architecture of SCZ, suggesting that the disorder is influenced by the combined effects of numerous common genetic variants. However, the translation of GWAS findings into clinically useful risk prediction models has been challenging.^[^ ^4^ ^]^ Genetic risk factors alone often have limited predictive power, as the complex pathogenesis of SCZ likely involves the interplay of various molecular mechanisms beyond genetic variations.^[^ ^5^, ^6^ ^]^

Transcriptomic analysis has emerged as a complementary approach to elucidate the molecular underpinnings of SCZ.^[^ ^7^, ^8^ ^]^ By examining disease‐driven gene expression patterns, researchers can uncover key genes and pathways involved in the pathogenesis of SCZ, which may also contain important genetic variations underlying disease susceptibility and development.^[^ ^9^, ^10^ ^]^ Recent biomedical research has opened new avenues for identifying disease‐associated features, particularly through the use of artificial intelligence techniques like machine learning (ML).^[^ ^11^ ^]^ While previous studies have employed ML on peripheral blood or prefrontal cortex (PFC) transcriptomic data to distinguish SCZ cases from healthy controls,^[^ ^12^, ^13^ ^]^ the absence of external validation and functional analysis on the identified genes has undermined reproducibility and limited their utility as stable disease‐responsive features. Furthermore, these studies are typically confined to either blood or PFC data, lacking an integrated approach that encompasses both peripheral and central transcriptomic profiles. This gap highlights the need for integrating PFC and peripheral blood transcriptomics via ML to uncover more stable disease‐responsive features and reliable peripheral biomarkers.^[^ ^14^ ^]^

**Figure 1:**
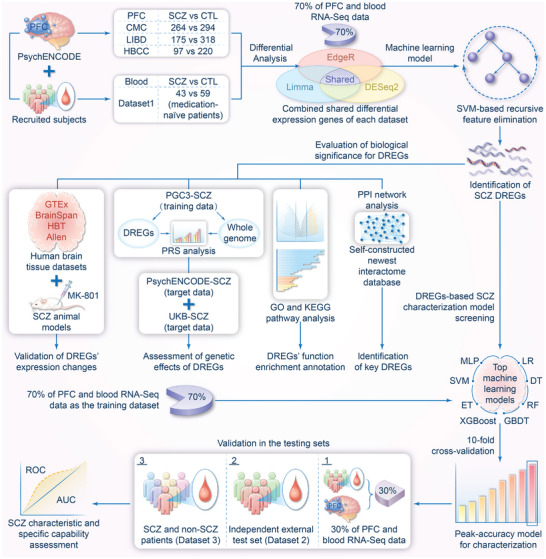
The workflow for SCZ DREGs identification, analysis, and characterization. (*SCZ DREGs identification*) Using PsychENCODE data (three PFC RNA‐Seq datasets) and new peripheral blood RNA‐Seq data, 70% were used for differential expression gene (DEG) analysis. DEGs in each dataset were identified by intersecting DESeq2, EdgeR, and Limma results, then subjected to support vector machine (SVM)‐based feature elimination to identify DREGs. (Biological significance analysis of *SCZ DREGs*) Protein–protein interaction analysis used a self‐constructed latest human interactome. GO and KEGG analyses revealed SCZ‐related pathway enrichment. DREGs expression were validated in human brain tissues and SCZ models. PRS analysis assessed DREGs' genetic contribution comparable to genome‐wide PRS. (Evaluation of *DREGs' SCZ characterization*) Eight top machine learning models performed tenfold cross‐validation on 70% of PFC and blood RNA‐Seq data to obtain the best characterization model. The best model was used to validate DREGs' SCZ characterization in three independent datasets: internal test set, external test set (Dataset 2), and SCZ/non‐SCZ patient set (Dataset 3). Results were evaluated using AUC values of ROC curves. SCZ: schizophrenia; PFC: prefrontal cortex; DREGs: disease‐responsive essential genes; PPI: protein–protein interaction; PRS: polygenic risk score; ROC: receiver operating characteristic; AUC: area under the curve; LR: logistic regression; DT: decision tree; RF: random forest; ET: extra tree; GBDT: gradient boosting decision tree; XGBoost: eXtreme gradient boosting; SVM: support vector machine; MLP: multilayer perceptron.

Building on GWAS insights, our study employs a comprehensive approach, integrating transcriptomic analysis with genomic data and experimental validation, to identify disease‐responsive essential genes (DREGs) that enhance SCZ characterization. By applying advanced ML methods to a large cohort of postmortem brain and peripheral blood RNA‐sequencing data, we aim to capture core SCZ‐driven transcriptional patterns, elucidate underlying biological mechanisms, and evaluate these DREGs as potential disease markers. To better illustrate our analytical framework and workflow, we have provided a detailed schematic in **Figure** **1**. Unlike previous studies focused on distinguishing SCZ cases from controls, we target disease‐driven molecular signatures involved in SCZ pathogenesis. This comprehensive approach aims to provide a deeper understanding of SCZ's complex molecular mechanisms and further develop improved characterization models with clinical applications. Our approach complements previous GWAS efforts and offers a fresh perspective on the disorder's genetic and genomic basis.

## 2 Results

### 2.1 Characterization of 184 SCZ DREGs

**Figure 2:**
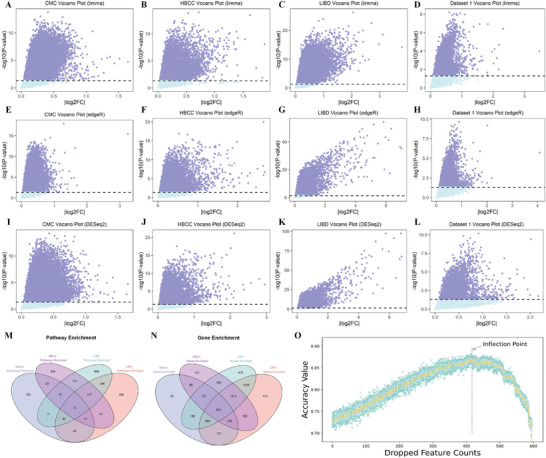
The results of characterization of 184 SCZ DREGs. (A–L) Differentially expressed genes in four datasets: Analysis of the differentially expressed genes obtained from four datasets (CommonMind Consortium [CMC], Human Brain Collection Core [HBCC], and Lieber Institute for Brain Development [LIBD], Dataset 1 from peripheral blood) using limma (A–D); edgeR (E–H) and DESeq2 (I–L). Given of the potential transcriptional heterogeneity of different tissues, the direction of expression changes was not strictly limited, and thus the absolute value of log2FC was considered. (M) 70 shared enriched pathways for differentially expressed genes: Presenting the 70 shared enriched pathways associated with differentially expressed genes in the four training sets. Each circle color represents pathways enriched in different training sets, with a significance threshold of *P*‐value <0.05. (N) 600 common genes included in the shared enriched pathways: Highlighting the 600 common genes found in the shared enriched pathways of differentially expressed genes across the four training sets. The circles denote genes included in the shared enriched pathways in different training sets, indicated by varying colors. (O) Result curve of recursive feature elimination: Demonstrating the result curve of recursive feature elimination based on the 600 shared genes. The x‐axis represents the number of discarded genes, while the y‐axis represents the average under the curve accuracy value of the SVM model after tenfold cross‐validation. The shaded area depicts the 95% confidence interval. Each point represents the result of a specific experiment, and the gray dotted line indicates the number of genes finally discarded when reaching the optimal AUC value.

Table S1 (Supporting Information) and **Figure** **2**A–L present the detailed results of differentially expressed genes by DESeq2, EdgeR, and Limma analysis. Pathway enrichment analysis results are shown in Table S2 (Supporting Information). Integrating pathways enriched with differentially expressed genes from the four training datasets identified 70 significant pathways and 600 corresponding genes (Figure 2M,N; Table S3, Supporting Information). Recursive feature elimination using the support vector machine (SVM) model (Figure 2O) selected 184 DREGs (Table S4, Supporting Information). These DREGs were further used for constructing a characterization model for SCZ.

### 2.2 DREGs Exhibit Significant Biological and Clinical Relevance

#### 2.2.1 A Significantly Interconnected Protein–Protein Interaction (PPI) Network Encoded by the DREGs

**Figure 3:**
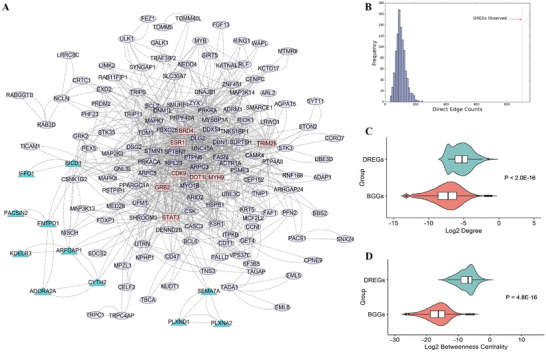
The characteristics of the significantly interconnected PPI Network encoded by the DREGs. (A) A densely interconnected PPI network encoded by DREGs: The visualization of a densely interconnected PPI network, where nodes represent genes and edges represent interaction relationships. Pink nodes indicate hub genes, while cyan nodes denote genes included in two functional modules. (B) Permutation test results of 1000 random PPI networks: During the permutation test, 1000 random PPI networks were generated by randomly selecting 155 genes from the human interactome to maintain the same number of nodes as the DREGs PPI network. The size of the direct connectivity component (number of edges in the network) was compared between the DREGs PPI network and the random PPI networks. The largest random PPI network had a direct connectivity component size of 248, which is significantly smaller than the observed 900 in the DREGs PPI network (grey dashed line). (C) Comparative box plots of node degree: A comparative box plot of the node degree between the DREGs PPI network and a background gene (BGG) PPI network. The Wilcoxon test was performed, revealing a highly significant difference between the two networks (*P* < 2 × 10^−16^). (D) Comparative box plots of betweenness centrality: A comparative box plot indicating the betweenness centrality between the DREGs PPI network and a BGG PPI network. The Wilcoxon test was conducted, showing a statistically significant difference between the two networks (*P* = 4.8×10^−16^). Data presented as violin plots with embedded box plots showing the distribution of log2‐transformed values. The box plots display the median (central line), first and third quartiles (box boundaries), and whiskers extending to the most extreme data points that are not considered outliers. The violin plots show the kernel density estimation of the underlying distribution for both DREGs and BGGs (background genes) groups.

We constructed a comprehensive human interactome dataset with 24 178 genes and 2 544 177 interactions (Table S5, Supporting Information). Among the 184 DREGs, we identified 155 with direct interactions, forming a densely interconnected PPI network of 155 genes with 900 interactions (**Figure** **3**A). Permutation tests compared this network to 1000 randomly generated PPI networks, showing that the DREGs PPI network had significantly more protein interactions (*P* < 1×10^−16^) (Figure 3B). Network parameters (node degree and betweenness centrality) indicated that DREGs had higher values than background (BG) genes (node degree *P* < 2×10^−16^, betweenness centrality *P* = 4.8×10^−16^) (Figure 3C,D). These findings demonstrate enriched protein interactions and central roles of DREGs in the network.

#### 2.2.2 Identification of 19 key DREGs in the PPI Network

**Figure 4:**
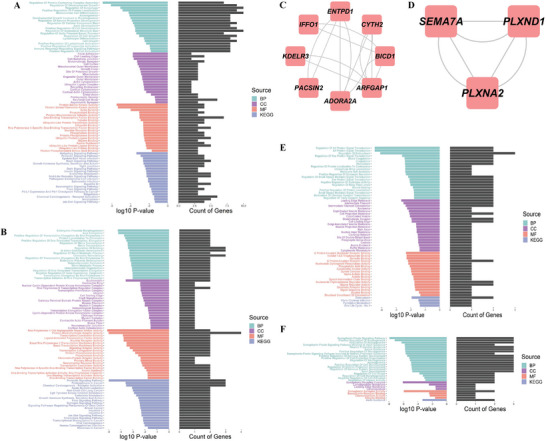
The results of dominant pathway enrichment in the DREG PPI network. (A) Pathway enrichment of top 32 genes in DREG set: Analysis of pathway enrichment for the top 32 genes in the DREG set. The presence of genes across different pathways (DREGs, Hub genes, Module 1, and Module 2) was examined with a repetition threshold of 30% of the maximum number of repetitions (135 times for *BCL*2, approximately 41 times for *TICAM1*). The left subfigure displays the significance of enriched GO (Molecular Function [MF], Biological Process[BP], Cell Component [CC]) and KEGG entries, focusing on the top 20 most significant entries. The right subfigure indicates the number of DREGs genes associated with each entry. Significant enrichments included immune response regulation, synaptic plasticity, neuronal development and projection, glutamatergic synapse, MAPK signaling pathway, JAK‐STAT signaling pathway, and neurotrophin signaling pathway. (B) Hub pathway enrichment of gene sets: Genes with direct interactions greater than 20 were extracted from the DREGs PPI network to identify hub genes. GO and KEGG enrichment analysis was conducted on the hub genes. The left subfigure presents the significance of GO (BP, CC, MF) and KEGG enriched entries, with the top 20 most significant entries. The right subfigure shows the gene count from the 184 DREGs present in each entry. (C) Function module 1: The specific genes enriched in this module are presented. (D) Function module 2: The specific genes enriched in this module are presented. (E) Module 1 pathway enrichment: Pathway enrichment analysis of the functional enrichment module 1. The left subfigure displays significant GO (BP, CC, MF) and KEGG entries, with the top 20 most significant entries. The right subfigure shows the gene count from the 184 DREGs present in each entry. (F) Module 2 pathway enrichment: Pathway enrichment analysis of the functional enrichment module 2. The left subfigure displays significant GO (BP, CC, MF) and KEGG entries, with the top 20 most significant entries. The right subfigure shows the gene count from the 184 DREGs present in each entry.

We analyzed the DREGs PPI network to investigate its characteristics. We defined hub genes as DREGs with at least 20 direct interactions with other DREGs, identifying 8 hub genes: *ESR1, GRB2, STAT3, BRD4, CDK9, TRIM28, MYH9*, and *DOT1L* (Figure 3A). Using ClusterONE, we identified two significant functional modules: module 1 (*P* = 0.019) and module 2 (*P* = 0.03) (**Figure** **4**C,D). Module 1 consists of 8 genes: *RFGAP1, CYTH2, ADORA2A, IFFO1, PACSIN2, ENTPD1, BICD1*, and *KDELR3*. Module 2 comprises 3 genes: *PLXND1, PLXNA2*, and *SEMA7A*.

#### 2.2.3 Dominant Enrichment of Pathways Associated with Synaptic Plasticity, Immune Inflammation, Neuronal Development, Neurotransmitters, and Astrocytes in the DREGs PPI Network

To examine the convergence of SCZ DREGs in the DREGs PPI network toward specific pathways, we performed GO and KEGG pathway enrichment analysis. The analysis identified significant pathways including synaptic plasticity, neuronal development/projection, synaptic transmission, inflammation regulation, calcium homeostasis, neurotransmitter regulation, vesicle transport/secretion, GPCR signaling, miRNA regulation, and *MAPK/neurotrophin/toll‐like receptor/TNF/JAK‐STAT* signaling (Table S6, Supporting Information). Further analysis of key DREGs, including 8 hub genes and 2 densely connected modules, revealed enrichments in pathways related to epigenetic gene regulation, immune response, inflammation, neurotransmitter secretion, synaptic transmission, astrocyte activation, synaptic plasticity, neuronal development, and N*otch/IL6/toll‐like receptor/JAK‐STAT/chemokine* signaling (Tables S7–S9, Supporting Information).

We further analyzed gene repetitions across different pathways within each gene set (Tables S10–S13, Supporting Information) to quantify pathway enrichment and assess the significance of each pathway. In the DREGs set, immune regulation, synaptic plasticity, neuronal development, glutamate synapse, and *MAPK/JAK‐STAT/neurotrophin* signaling were significantly enriched (Figure 4A). The hub gene set showed remarkable enrichments in chromatin remodeling, transcriptional regulation, miRNA regulation, *JAK‐STAT* signaling, and chemokine signaling (Figure 4B). Module 1 was associated with glutamate‐based neurotransmitter secretion and synaptic transmission (Figure 4E), while module 2 was related to synaptic plasticity, neuronal development, and projection (Figure 4F). Notably, the most repeated genes in the hub and module gene sets were among the top 32 genes in the DREGs set (Tables S10–S13, Supporting Information). Additionally, *SYT11*, like *ADORA2A*, is another noteworthy gene linked to SCZ in our unpublished study, playing a role in mediating SCZ‐like behaviors through dopamine overtransmission.

#### 2.2.4 Expression Changes in DREGs Across Various Human Brain Tissues

**Figure 5:**
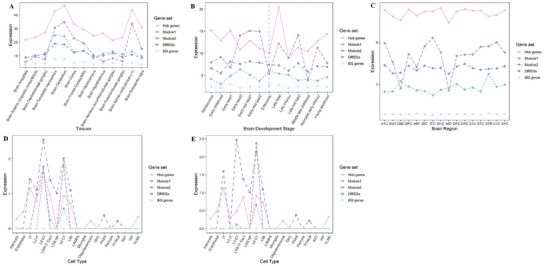
The results of expression patterns in DREGs across various human brain tissues. (A) Expression patterns of DREGs, key DREGs, and BG genes in 13 types of human brain tissues in the GTEx database. The expression trends of hub genes and genes in module 2 were consistent across different brain tissues, showing high expression in the brain spinal cord (vervical c‐1). On the other hand, genes in module 1 exhibited low expression in this specific brain tissue. (B) Spatiotemporal expression patterns of DREGs, key DREGs, and BG genes in 13 brain development stages in the Brainspan database. These stages ranged from embryonic to young adulthood. BG genes and module 1 genes showed no significant changes in expression throughout development (*P* = 0.1146328, one‐way repeated measures ANOVA). However, hub genes and module 2 genes displayed noticeable expression fluctuations, with hub genes peaking after birth and maintaining relatively high expression levels during development. (C) Expression patterns of DREGs, key DREGs, and BG genes in 16 brain regions in the HBT database. The 16 brain areas include primary auditory (A1) cortex (A1C), amygdala (AMY), cerebellar cortex (CBC), dorsolateral prefrontal cortex (DFC), hippocampus (HIP), posterior inferior parietal cortex (IPC), inferior temporal cortex (ITC), primary motor (M1) cortex (M1C), mediodorsal nucleus of the thalamus (MD), medial prefrontal cortex (MFC), orbital prefrontal cortex (OFC), primary somatosensory (S1) cortex (S1C), superior temporal cortex (STC), striatum (STR), primary visual (V1) cortex (V1C), and ventrolateral prefrontal cortex (VFC). Hub genes displayed the highest overall expression across all regions, while genes in module 2 exhibited significant expression variations among brain regions. (D) Expression patterns of DREGs, key DREGs, and BG genes in cell types of the middle temporal gyrus in the Allen database. (E) Expression patterns of DREGs, key DREGs, and BG genes in cell types of the anterior cingulate gyrus in the Allen database. In both brain regions, the key DREGs in glutamate‐type neurons (IT, L4 IT, L5 ET, L5/6 IT Car3, L6 CT, L6b) exhibited obvious expression fluctuations, along with consistent expression trends in hub genes and DREGs. Furthermore, the expression patterns of genes in modules 1–2 varied between the two brain regions. In the middle temporal gyrus, genes in modules 1–2 were mainly expressed in the cell type L6 CT, while in the anterior cingulate gyrus, genes in modules 1–2 were highly expressed not only in the cell type L6 CT but also in L5 ET. All values are presented as mean expression levels across genes within each gene set (Hub genes, Module1, Module2, DREGs, and BG genes) for each condition (tissues, developmental stages, brain regions, or cell types). Expression values were normalized and processed according to their respective databases: TPM values for GTEx, RPKM values for BrainSpan, and normalized expression values for HBT and Allen Brain Atlas data.

We analyzed RNA‐seq data from various sources to study the expression patterns of DREGs in different brain contexts. In human brain tissues (GTEx V8 database), DREGs showed higher expression levels compared to BG genes (DREGs: *P* = 0.085, hub genes: *P* < 1×10^−8^, module 1: *P* = 0.020, module 2: *P* = 0.001), with hub genes displaying the highest overall expression (**Figure** **5**A). Hub genes and genes in module 2 showed consistent expression trends across different brain tissues, while genes in module 1 had lower expression in certain brain regions (Figure 5A). During brain development (BrainSpan database), DREGs, hub genes, and module 2 genes showed significantly higher expression levels across all developmental stages compared to BG genes (DREGs: *P* = 2×10^−5^, hub genes: *P* < 1×10^−8^, module 2: *P* < 1×10^−8^) (Figure 5B). Hub genes exhibited a peak in expression after birth, while hub genes and genes in module 2 displayed prominent expression fluctuations throughout development. In diverse brain regions (Human Brain Transcriptome [HBT] database), DREGs, hub genes, and module 1–2 genes had significantly higher expression levels across different brain regions (all *P* < 1×10^−16^) (Figure 5C). Hub genes consistently showed the highest expression, and module 2 genes demonstrated expression variations specific to different brain regions. In the SCZ‐associated middle temporal gyrus (MTG) and anterior cingulate gyrus (CgGr) (Allen database), DREGs, hub genes, module 1–2 genes showed significantly higher expression levels compared to BG genes (Figure 5D,E). Fluctuating expression patterns of key DREGs were observed in glutamate‐type neurons, with consistent trends between hub genes and DREGs. Notably, genes in modules 1 and 2 exhibited distinct expression patterns between MTG and CgGr, indicating diverse roles in different neuron types, particularly glutamatergic neurons.

#### 2.2.5 Significant Changes in Expression Patterns of 9 Novel Key DREGs in Animal Models

**Figure 6:**
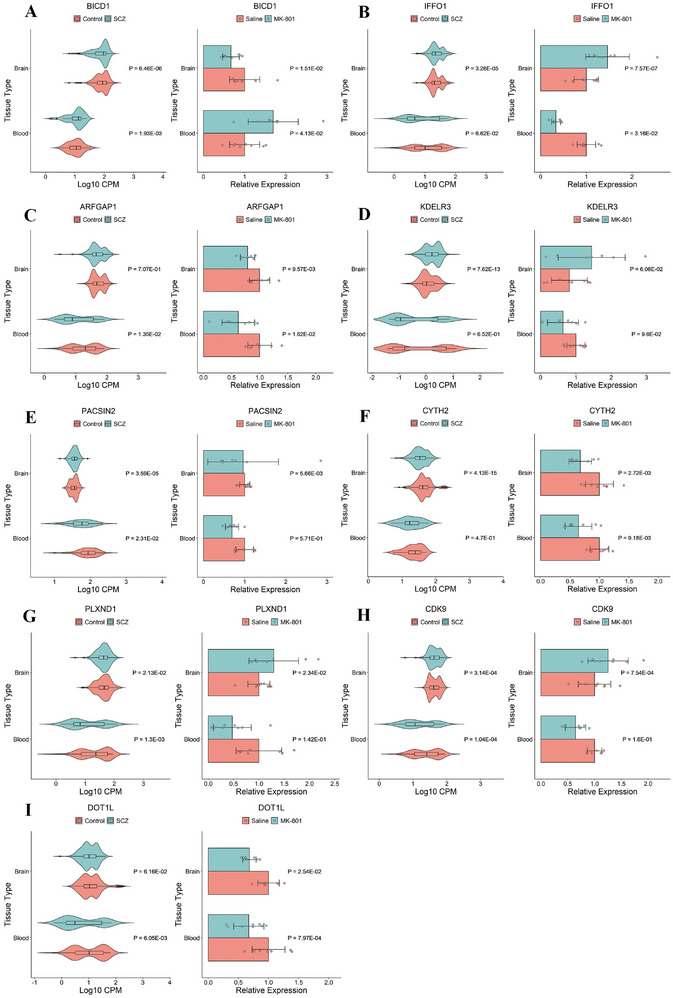
Differential expression profiles of 9 key DREGs in the peripheral blood and prefrontal cortex of SCZ animal models and human RNA‐seq datasets. mRNA expression changes of (A) *BICD1*; (B) *IFFO1*; (C) *ARFGAP1*; (D) *KDELR3*;(E) *CYTH2*; (F) *PACSIN2*; (G) *PLXND1*; (H) *CDK9*; (I) *DOTIL* in the peripheral blood and prefrontal cortex of human (left) and mice (right). The human peripheral blood data is derived from the combined Datasets 1 and 2, while the human prefrontal cortex data is obtained from the merged datasets of the CMC, LIBD, and HBCC. Statistical comparisons were performed using Student's t‐test. Data are presented as means ± SEM (n = 8 per group). Significant differences between SCZ and control groups are indicated in the figure (*P* < 0.05).

We identified 19 key DREGs, including 8 hub genes, 8 genes from module 1, and 3 genes from module 2. Among these, 10 genes (*ADORA2A, ENTPD1, PLXNA2, SEMA7A, ESR1, GRB2, STAT3, BRD4, TRIM28, MYH9*) have previously been associated with SCZ (see Supporting Information). To validate the expression patterns of these key DREGs, we focused on 9 novel genes (*BICD1, IFFO1, ARFGAP1, KDELR3, CYTH2, PACSIN2, PLXND1, CDK9, DOT1L*) using an SCZ animal model induced by MK‐801 (Figure S1, Supporting Information). We examined the mRNA levels of these 9 key DREGs in the peripheral blood and the PFC of the modeled mice (**Figure** **6**). In the animal models, 8 out of the 9 novel genes showed statistically significant expression changes in the brain samples, with the *KDELR3* gene exhibiting a trend toward significance (Figure 6A–I). Although four DREGs (*KDELR3, PACSIN2, CDK9, PLXND1*) did not exhibit statistically significant differences in the peripheral blood of the SCZ animal model, their expression trends were consistent with those observed in human brain data (Figure 6D,E,G,H). Despite small sample sizes, these findings confirm DREGs as reliable SCZ responsive indicators, with potential to elucidate SCZ mechanisms.

### 2.3 Strong Polygenic Risk for SCZ Associated with DREGs Polygenic Risk Scoring (PRS)

When applied to the UK Biobank (UKB)‐SCZ dataset, both the genome‐wide PRS and the 184‐DREGs PRS derived from Psychiatric Genomic Consortium version 3 (PGC3)‐SCZ showed significant associations with SCZ status (permutation *P* < 0.00001; Table S14 and Figure S2, Supporting Information). In the smaller PsychENCODE‐SCZ dataset, the genome‐wide PRS remained significant (permutation *P* < 0.00001), although the significance of the 184‐DREGs PRS was slightly reduced (permutation *P* < 0.005). However, the consistent effect direction with odds ratios (ORs) > 1.1 was noteworthy (Table S14, Supporting Information). These findings suggest that the polygenic risk contributed by the 184‐DREGs SNPs for SCZ is significantly higher than random chance and is even comparable to those optimal PRS using genes across whole genome.

### 2.4 DREGs Exhibit Characteristic Capabilities and Specificity for SCZ

**Figure 7:**
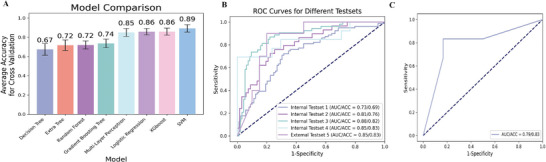
Assessment of characteristic performance and specificity of DREGs for SCZ. (A) Performance evaluation results of optimized machine learning models: Results of 10‐fold cross‐validation on 8 optimized machine learning models using the combined training dataset. The x‐axis represents the names of the 8 optimized machine learning models, while the y‐axis represents the average accuracy achieved through 10‐fold cross‐validation. All values are presented as mean ± SEM of accuracy values obtained from 10‐fold cross‐validation. (B) Receiver operating characteristic (ROC) curves of DREG‐based SVM model for different testsets: ROC curves illustrating the performance of the DREG‐based SVM model on various test sets.(C) ROC curves for differentiating SCZ and non‐SCZ patients: The ROC curve demonstrating the ability to distinguish between patients with SCZ and patients with non‐SCZ conditions. Only 1 out of 6 SCZ patients was misclassified, and only methamphetamine‐induced psychosis was not differentiated among the 6 non‐SCZ diseases. The ROC curves, AUC (area under the curve (AUC), and ACC (accuracy) values were calculated using the scikit‐learn package in Python. Specifically, the roc_curve and auc_score functions were used for ROC curve generation and AUC calculation, while accuracy_score was used for ACC calculation.

To optimize characterization models for SCZ, we combined four training sets and evaluated eight models using tenfold cross‐validation, with optimized parameters detailed in Table S15 (Supporting Information). The SVM model performed best, surpassing other models with an average accuracy of 89.21% (**Figure** **7**A). We selected the optimized SVM model, called the DREGs‐based SVM (DRES) model, as the ideal characterization model. Testing the DRES model on internal datasets showed accuracy rates of 69%, 76%, 82%, and 83%, with corresponding area under the curve (AUC) values of 73%, 81%, 88%, and 85% (Figure 7B). External evaluation on testset Dataset 2 demonstrated a characteristic accuracy of 83% and an AUC value of 85% (Figure 7B). The DRES model effectively differentiated SCZ from non‐SCZ conditions, achieving an AUC of 79% and an accuracy of 83% (Figure 7C). Our findings suggest the potential of the DRES model in identifying individuals with SCZ across different disease categories.

## 3 Discussion

This study leverages cross‐tissue transcriptomic data and various omics annotation/integration approaches to provide both biological and clinical insight on SCZ manifestation. In clinical settings, there is currently a lack of effective approaches for diagnosing and characterizing SCZ. This study utilizes ML‐based approaches with RNA‐seq datasets to characterize SCZ. Our novel methods address the limitations of current characterization methods (low discriminative ability), further improving characterization for individuals with SCZ (AUC > 0.8). Analyzing the PPI network, performing pathway enrichment, utilizing human brain datasets, and conducting laboratory experiments collectively demonstrate the crucial role of DREGs in SCZ etiology. This approach has potential for extending to other neuropsychiatric disorders, facilitating precision psychiatry.

While many studies have utilized transcriptomics data and ML to identify characteristic expression patterns^[^ ^15^ ^]^ or biomarkers for SCZ,^[^ ^16^ ^]^ there remains room for further refinement. Most of these studies did not conduct functional analyses or experimental validations of the identified genes,^[^ ^12^, ^13^, ^15^, ^16^ ^]^ which may limit their effectiveness in providing stable disease characterizations. In our study, we first combined traditional bioinformatics methods and recursive feature elimination algorithms with multiple RNA‐seq datasets. This approach identified 184 SCZ DREGs, improving our ability to extract relevant disease‐responsive features. Then through PPI network analysis, we found strong evidence that DREGs form a highly interconnected network involved in SCZ pathogenesis. Within this network, we identified 19 key DREGs, including 11 genes in densely connected modules and 8 hub genes. Enrichment analysis revealed shared gene ontology terms and pathways, including neuronal development, immune response regulation, synaptic plasticity, and epigenetic gene expression regulation, known to be involved in SCZ.^[^ ^17^ ^]^ Both the genome‐wide PRS and the 184‐DREGs PRS were significantly associated with SCZ status, validating the biological relevance of DREGs in SCZ characterization. These findings support the use of DREGs as a reliable gene set for characterizing SCZ.

Among the 19 key DREGs, 10 have previously been linked to SCZ. *SYT11*, along with *ADORA2A*, is notable as both genes play crucial roles in the glutamatergic and dopaminergic systems,^[^ ^18^ ^]^ which are implicated in SCZ pathogenesis.^[^ ^17^ ^]^ *ADORA2A* in astrocytes regulates glial glutamate transporter 1 activity, potentially leading to disturbances in glutamine levels and SCZ induction.^[^ ^19^ ^]^ Besides, our unpublished study suggests that abnormal *SYT11* expression contributes to SCZ‐related behaviors through dopamine overtransmission. Significant associations between *ADORA2A*, *SYT11* polymorphisms, and SCZ susceptibility have been identified, indicating their central role in triggering SCZ.^[^ ^20^ ^]^ Additionally, nine newly identified genes (*BICD1*, *IFFO1*, *ARFGAP1*, *KDELR3*, *CYTH2*, *PACSIN2*, *PLXND1*, *CDK9*, *DOT1L*) are implicated in SCZ, some of which are involved in other central nervous system disorders.^[^ ^21^ ^]^ Furthermore, all nine of these novel genes are involved in synaptic function (*PACSIN2*,^[^ ^22^ ^]^ *PLXND1*,^[^ ^23^ ^]^ *DOT1L*,^[^ ^24^ ^]^) neurodevelopment (*BICD1*,^[^ ^25^ ^]^ *ARFGAP1*,^[^ ^26^ ^]^ *CYTH2*,^[^ ^27^ ^]^) and immune response (*IFFO1*,^[^ ^28^ ^]^ *KDELR3*,^[^ ^29^ ^]^ *CDK9*,^[^ ^30^ ^]^), aligning closely with our enrichment analysis. These findings resonate with the conclusions of a recent Science article, which used single‐nucleus RNA sequencing technology to identify significant transcriptional changes in synaptic and neurodevelopmental pathways across various cell types in the PFC of SCZ patients.^[^ ^31^ ^]^ This convergence of evidence further emphasizes the critical roles of these key DREGs in synaptic plasticity and neuronal development in the pathophysiology of SCZ. Additionally, RT‐qPCR validation revealed consistent similar expression change directions for these nine key DREGs between human and SCZ animal model brain samples, with statistically significant changes observed in both. Peripheral blood samples showed consistent trends across humans and animal models, though not all reached statistical significance. These findings highlight the potential relevance of these genes in SCZ pathophysiology, demonstrating parallels in expression patterns between human and animal models in both central and peripheral tissues. Further functional research is needed to understand how these nine genes regulate molecular mechanisms in SCZ.

Transcriptomic data poses challenge due to its wide nature and the potential for overfitting in data analysis models.^[^ ^32^ ^]^ To address this, we employed dimensionality reduction and utilized a practical ML model to minimize overfitting.^[^ ^33^ ^]^ Our unique analysis methods identified SCZ DREGs from diverse RNA‐seq datasets (brain and blood) and provided insights into underlying biological processes and pathways. We also developed an accurate disease characterization model using DREGs and ML, indicating strong performance in classifying SCZ patients and distinguishing them from other psychiatric disorders. While previous studies using omics data have improved risk stratification for SCZ and other psychiatric disorders,^[^ ^34^ ^]^ our pipeline offers a more targeted focus by characterizing SCZ‐responsive genes and identifying core pathogenic mechanisms with minimal transcriptome data. Unlike the broad approach of Wang et al.,^[^ ^34^ ^]^ which created a comprehensive functional genomic resource, our study focuses on disease‐driven expression patterns specific to SCZ. By leveraging ML, functional annotation, network analysis, and animal validation, we provide deeper insights into the roles of DREGs in synaptic function, immune regulation, and neurodevelopment. Notably, the disease‐responsive essential genes may contain SCZ risk or development variations, but examining how these SNPs regulate DREGs' expression changes was beyond the scope of our study.

Our study addresses some limitations in previous research. Merikangas et al.^[^ ^35^ ^]^ faced methodological inconsistencies and covariate variations, leading to few consistently replicated genes. Unlike their literature‐based approach, we integrate multiple RNA‐seq datasets with ML, ensuring the stability of identified genes through experimental validation. By aligning with LIBD principles,^[^ ^36^ ^]^ we correct for batch effects and include critical covariates such as age and sex, addressing confounding factors and improving robustness. While there may be ancestry‐dependent differential expression genes for brain disorders, most are less constrained and sensitive to evolutionary changes.^[^ ^37^ ^]^ However, by conducting differential expression analyses independently for each dataset and employing ancestry‐matched data in our PRS analysis, we enhance the validity and reliability of our findings. This approach ensures that ancestry‐related biases are unlikely to significantly impact our results. Despite fewer peripheral blood samples, our findings remain robust through repeat validation. Additionally, recent findings by Ruzicka et al.^[^ ^31^ ^]^ support the importance of neurodevelopment and synapse‐related pathways in SCZ, validating our mechanisms. We also evaluated the genetic effects of DREGs via PRS analysis, offering new insights into SCZ's genetic basis. While Wang et al.^[^ ^34^ ^]^ provided a broad foundational understanding, our study offers targeted mechanistic insights and potential clinical applications, emphasizing the unique advantages of our approach. Our work demonstrates the importance of LIBD principles^[^ ^36^ ^]^ like sample size, covariates, and expression complexity, reflecting the effectiveness of these principles in advancing SCZ research.

While our study demonstrates robustness in identifying SCZ characterization using RNA‐seq data and ML techniques, caution should be exercised in extrapolating the findings to broader populations. First, gene expression changes are regulated by a multitude of factors and cannot be solely attributed to disease response. Second, the relatively small sample size and the predominance of Han Chinese participants may limit the generalizability of our results to other ethnic groups or clinical settings. Additionally, the reliance on peripheral blood samples for RNA‐seq analysis may not fully capture the disease‐specific transcriptional changes occurring in the brain, and the absence of regulatory RNA data, such as miRNA, limits our understanding of the transcriptional regulatory networks associated with disease features. The lack of unified processing standards across different laboratories adds complexity and challenges to our study. Despite these limitations, our model offers significant value in accurately characterizing SCZ. The findings have important implications for clinical practice, potentially aiding in earlier and more precise diagnosis. Give of our preliminary results, future studies, with larger sample sizes, diverse populations, and additional types of data, would be required to further validate and expand our findings.

## 4 Conclusion

In summary, our study presents a comprehensive approach to enhance SCZ characterization by integrating ML‐based transcriptomic analysis with genomic data annotation and experimental validation. We identified 184 DREGs significantly associated with SCZ, conducted pathway enrichment and PPI network analyses, and validated key DREGs in SCZ animal models. Additionally, we assessed the genetic contribution of DREGs using PRS and developed high‐performance machine‐learning models for SCZ characterization. Our findings contribute to improved disease characterization, elucidate SCZ molecular mechanisms, and suggest new potential therapeutic targets. Future research will focus on functional validation, longitudinal studies, and expanding to broader cohorts to enhance robustness and generalizability.

## 5 Experimental Section

### Sample Collection

Participants were recruited from multiple sites. Dataset 1 included episodic SCZ patients and healthy controls from Yingtan Mental Health Hospital. Dataset 2 comprised episodic SCZ patients from Shandong Mental Health Center and healthy controls from Qilu Hospital of Shandong University. Dataset 3 consisted of both episodic SCZ and non‐SCZ psychiatric patients from Yingtan Mental Health Hospital. Participants provided written informed consent and 2.5 mL of whole blood was collected for RNA sequencing. The study followed ethical principles outlined in the 2002 Declaration of Helsinki, with approval from the Medical Ethics Committee of Xi'an Jiaotong University Health Science Center. The study employed three independent datasets (Dataset 1 with 43 SCZ and 59 controls; Dataset 2 with 10 SCZ and 20 controls; Dataset 3 with 6 SCZ and 6 non‐SCZ psychiatric patients), with Supplementary Methods (Supporting Information) providing detailed information and inclusion/exclusion criteria. All patient samples had more than one‐month medication‐free history, and Dataset 1 consisted of medication‐naïve first‐episode patients. This study was conducted in accordance with the ethical principles outlined in the 2002 Declaration of Helsinki. The protocol was approved by the Medical Ethics Committee of Xi'an Jiaotong University Health Science Center (approval number: NO. 2017030). Written informed consent was obtained from all participants prior to their enrollment in the study.

### RNA Sequencing and Data Pre‐Processing

Total RNA extraction from peripheral blood samples was performed using the PAXgene Blood RNA Kit (BD Biosciences, USA) following the manufacturer's instructions for datasets 1, 2, and 3. Total RNA quality was assessed using agarose gel electrophoresis and quantified with a NanoDrop spectrophotometer (NanoDrop, USA). For mRNA library construction, total RNA underwent ribosomal RNA depletion using the Epicenter Ribo‐Zero kit. TruSeq RNA Sample Preparation kit processed 3 µg RNA/sample following Illumina's protocol. RT‐PCR employed Phusion high‐fidelity DNA polymerase, indexed (X) primers, and universal PCR primers. AMPure XP system purified the products, while library quality was evaluated on the Agilent Bioanalyzer 2100 system. The Illumina NovaSeq 6000 platform sequenced the mRNA libraries. Initial quality control was performed using FastQC^[^ ^38^ ^]^ to assess sequencing data quality, including base quality distribution, GC content, and sequence duplication levels. Fastp^[^ ^39^ ^]^ software was used to filter out low‐quality reads and adapter sequences. The remaining reads were aligned to the human reference genome hg19 using HISAT2.2.4.^[^ ^40^ ^]^ All count data were finally generated for subsequent analysis.

### Existing Data and Combined Data Preparation

This study also utilized three PFC RNA‐seq datasets (CommonMind Consortium [CMC], Human Brain Collection Core [HBCC], and Lieber Institute for Brain Development [LIBD]) accessed from PsychENCODE, comprising SCZ patients and healthy controls (Table S16, Supporting Information). To ensure reliable results, these datasets and Dataset 1 (43 SCZ and 59 controls) were split into training and internal test sets (7:3 ratio, no overlap). The training sets were used for DREG extraction, model training, and hyperparameter optimization, while the internal test sets assessed model performance. Dataset 2 served as an external test set to evaluate the analysis pipeline and findings' robustness. PRS was performed using three large‐scale genetic datasets (PGC, UKB, and PsychENCODE project including CMC, HBCC, and LIBD, detailed in Table S16, Supporting Information) to assess SCZ status holistically. PGC version 3 data for SCZ GWAS was publicly available. UKB raw data was accessed under approved application No. 86920. PsychENCODE data was accessed via Synapse portal with granted approval to Dr. Guan's team. This approach aimed to evaluate the collective impact of identified SCZ DREGs and their genomic contribution to this mental disorder. Supplementary Methods (Supporting Information) provide brief descriptions of each dataset.

### Extraction of Characteristic DREGs

The four RNA‐seq datasets (CMC, HBCC, LIBD, Dataset 1) were divided into training and test sets (7:3 ratio). The SVA (Surrogate Variable Analysis) package was used to correct for batch effects, ensuring that the variability introduced by different batches did not confound our results. Differential expression analysis was performed on the training datasets using the limma^[^ ^41^ ^]^, Deseq2^[^ ^42^ ^]^, and edgeR^[^ ^43^ ^]^ packages, with significance determined as *P*‐value < 0.05 and |logFC| > 0. To mitigate the impact of age and gender on gene expression, these variables were included as covariates in the differential expression analysis using DESeq2, edgeR, and limma. Specifically, the design matrix incorporated age and gender along with the primary condition of phenotype (SCZ vs. control). The final set of differentially expressed genes for each training dataset was determined by taking the intersection of the results from these three software packages. ENSEMBL IDs were converted to ENTREZ IDs, and pathway enrichment analysis was conducted using the clusterProfiler package.^[^ ^44^ ^]^ Significant pathways (*P*‐value < 0.05) and their shared differentially expressed genes were integrated to identify responsive characteristics for SCZ. After correcting and standardizing the count matrices, recursive feature elimination (RFE)^[^ ^45^ ^]^ with a SVM model was employed to identify characteristic DREGs. Further details can be found in the Supplementary Methods (Supporting Information).

### Analysis of the Biological Basis of DREGs

The newest human interactome database was self‐constructed by integrating PPI data from multiple sources (String, Biogrid, Bioplex, CCSB, HINT, HPRD, IntAct, and Mint) and analyzed the PPI networks formed by DREGs. Various network parameters were computed, including node degree and betweenness centrality, to understand the characteristics of DREGs in the network. Permutation tests were performed to compare the network connectivity with 1000 randomly generated networks, while Wilcoxon tests were used to compare network parameters (node degree and betweenness centrality) between DREGs and background genes. Hub genes and densely connected modules were identified in the PPI network, defining all hub genes and those within these modules as key DREGs. Pathway enrichment analysis explored the biological functions of DREGs, hub genes, and modules. Additionally, gene expression profiles in various tissues, developmental stages, brain regions, and specific cell types were analyzed using RNA sequencing data from multiple databases (GTEx, BrainSpan, HBT, and Allen). These analyses provided insights into the functional roles and expression patterns of DREGs relevant to SCZ. Further details can be found in the Supplementary Methods (Supporting Information).

### Detecting Expression Patterns of Key DREGs in SCZ Animal Models

All animal experiments were performed using male C57BL/6J mice (*n* = 8 per group) obtained from Beijing Vital River Laboratory Animal Technology Co., Ltd. (Beijing, China). The experimental procedures were conducted in accordance with institutional guidelines and approved by the Institutional Animal Care and Use Committee of Xi'an Jiaotong University (approval number: No. 2022680). SCZ mice models were established by NMDA receptor antagonist MK‐801 to further validate the expression changes of SCZ DREGs. Then the prefrontal cortex and blood samples were collected for RNA extraction. The following qRT‐PCR were carried out in the Bio‐Rad CFX96 detection instrument (Bio‐Rad, USA). Primer sequences are provided in Table S17 (Supporting Information). For more details, please refer to the Supplementary Methods (Supporting Information).

### Assessing the Genetic Effect of DREGs Using PRS

To assess the polygenic risk of SCZ, PRSice2 was employed with GWAS summary statistics (PGC3) and raw data (PsychENCODE‐SCZ and UKB‐SCZ) with matched ancestry. The aim was to characterize SCZ risk in PsychENCODE‐SCZ or UKB‐SCZ (target data), using PGC3‐SCZ as the training dataset.^[^ ^46^ ^]^ No sample overlap occurred between the training and target datasets. Common SNPs (minor allele frequency > 0.05) in both datasets were analyzed for compatibility. Exclusion criteria included imputation scores < 0.5 in training data and palindromic SNPs with ambiguous alleles. Two PRS models were created: one with genome‐wide SNPs and another mapping to DREGs (± 10kb boundary). PRS were standardized and associated using logistic regression, adjusting for gender and principal components (3 for PsychENCODE‐SCZ, 4 for UKB‐SCZ). PRSice2 generated optimal PRS scores across different *P* thresholds (5×10^−8^, 10^−7^, 10^−6^, 10^−5^, 10^4^, .001, .01, .05, 0.1, 0.2, 0.3, 0.4, 0.5, and 1),^[^ ^47^ ^]^ with permutation (100 000 times) correcting for multiple testing and overfitting.^[^ ^48^ ^]^

### SCZ Characterization via ML Models

Four training sets of RNA‐seq data were combined to create a unified dataset and employed eight high‐performance ML models. These models were used to assess the reliability and stability of DREGs in characterizing SCZ, including logistic regression (LR), decision tree (DT), random forest (RF), extra tree (ET), gradient boosting decision tree (GBDT), eXtreme gradient boosting (XGBoost), SVM, and multilayer perceptron (MLP). The model with the best generalization performance was selected after hyperparameter tuning and validated it using four internal test sets and one external test set (Dataset 2). The model's discriminative performance between SCZ and non‐SCZ disorders was also evaluated using Dataset 3. The model performance was evaluated using the scikit‐learn package in Python to calculate the AUC and accuracy values. Supplementary Methods (Supporting Information) provide further details.

### Statistical Analysis—Pre‐Processing

RNA‐seq data underwent quality control using FastQC v0.11.9, with low‐quality reads and adapter sequences filtered by Fastp v0.20.0. Batch effects were corrected using SVA package. Data normalization included variance stabilizing transformation in DESeq2. Age and gender were included as covariates in differential expression analyses, with data aligned to human reference genome hg19 using HISAT2.2.4.

### Statistical Analysis—Data Presentation

Laboratory experimental data and animal experiments are presented as mean ± SEM. RNA‐seq expression values are presented according to their respective databases (TPM values for GTEx, RPKM values for BrainSpan, and normalized expression values for HBT and Allen Brain Atlas). Network analysis results are presented as violin plots with embedded box plots showing log2‐transformed values, with PPI network comprising 155 DREGs forming 900 direct interactions. Machine learning model performance is presented as mean ± SEM from tenfold cross‐validation.

### Statistical Analysis—Sample size

The study analyzed RNA‐sequencing data from two sources: 1) PsychENCODE public database (536 SCZ patients, 832 controls), and 2) the newly generated peripheral blood RNA‐seq data from three independent cohorts (Dataset 1: 43 SCZ and 59 controls; Dataset 2: 10 SCZ and 20 controls; Dataset 3: 6 SCZ and 6 non‐SCZ psychiatric patients). Animal experiments used 8 mice per group.

### Statistical Analysis—Statistical Methods

Differential expression analysis employed three algorithms (limma, DESeq2, and edgeR) with significance defined as P‐value < 0.05 and |logFC| > 0. PPI network connectivity was evaluated using two‐sided permutation tests (1000 times), while network parameters were compared using two‐sided Wilcoxon tests. One‐way repeated measures ANOVA assessed expression changes across developmental stages. Student's *t*‐test was used for animal experimental data. Pathway enrichment used hypergeometric tests with P‐value < 0.05 threshold. PRS analysis used permutation testing (100 000 times) for multiple testing correction.

### Statistical Analysis—Software

RNA‐seq analyses were performed using R version 4.2.0 with packages including limma, DESeq2, edgeR, and clusterProfiler. Network analyses used R packages igraph and ggplot2. Machine learning analyses were conducted using Python's scikit‐learn package, with roc_curve and auc_score functions for receiver operating characteristic (ROC) curves and AUC calculations, and accuracy_score for ACC calculations. PRS analyses used PRSice2. Animal experimental data were analyzed using SPSS version 23.

## Conflict of Interest

No. The authors declare no conflict of interest.

## Supporting information



Supporting Information

## Data Availability

The data that support the findings of this study are available on request from the corresponding author. The data are not publicly available due to privacy or ethical restrictions.
