# Supplementary material for: Integrated Transcriptome Analysis Reveals Novel Molecular Signatures for Schizophrenia Characterization
Source: Adv Sci (Weinh). 2024 Nov 20;12(2):2407628. doi: 10.1002/advs.202407628 (PMC11727269; doi:10.1002/advs.202407628)
Supplement: Supplementary file 1 — Supporting Information [file ADVS-12-2407628-s001.docx]

**Supporting Information**

*Tong Ni, Yu Sun, Zefeng Li, Tao Tan, Wei Han, Miao Li, Li Zhu, Jing Xiao, Huiying Wang, Wenpei Zhang, Yitian Ma, Biao Wang, Di Wen, Teng Chen, Justin Tubbs, Xiaofeng Zeng*, Jiangwei Yan *, Hongsheng Gui *, Pak Sham , Fanglin Guan**

*Tong Ni, Yu Sun,, Zefeng Li, and Tao Tan contributed equally to this work.*

**Corresponding authors.*

Contents:

Supplementary Methods

Supplementary Tables (S1-17)

Supplementary Figures (S1-2)

Supplementary References

**Supplementary Methods**

**1. Subjects**

For Dataset 1, 2 and 3, the consensus diagnoses of schizophrenia (SCZ) were made by two senior psychiatrists based on the Diagnostic and Statistical Manual of Mental Disorders, 4th Edition. To ensure the accuracy of the study, certain criteria were put in place for participant selection. Patients were excluded if they had a history of substance abuse, suicidality, abnormal laboratory results or electrocardiogram/electroencephalogram readings, brain surgery, unstable medical condition, viral infection, or if they had taken antipsychotic medications within the month prior to recruitment. Healthy controls, on the other hand, were individually interviewed using the Structured Clinical Interview for DSM-IV-TR Axis I Disorders Nonpatient Version (SCID-NP) to ensure the absence of any psychiatric disorder. They also reported no personal or family history of physical illness or mental illness.

Dataset 1: consisted of 43 SCZ patients and 59 gender- and age-matched healthy controls, used for identifying DREGs and establishing SCZ characterization models.

Dataset 2: included 10 SCZ patients and 20 healthy controls, matched in terms of gender and age, to assess the reliability and consistency of DREGs and the novel SCZ characterization model.

Dataset 3: contained 6 SCZ patients and 6 patients with other psychiatric disorders (autism, depression, attention deficit hyperactivity disorder, bipolar mania, bipolar depression, methamphetamine-induced psychosis), used to investigate the specificity of DREGs and the novel model in characterizing SCZ. Detailed inclusion and exclusion criteria for non-SCZ patients in this dataset were as follows.

Autism

Inclusion criteria:

Confirmed diagnosis of Autism Spectrum Disorder (ASD) based on DSM-5 or ICD-10 criteria.

Exclusion criteria:

Presence of other primary psychiatric disorders (e.g., schizophrenia, bipolar disorder).

Severe intellectual disability or language impairment that hinders participation.

Depression

Inclusion criteria:

Diagnosis of major depressive disorder (MDD) or persistent depressive disorder (PDD) according to DSM-5 criteria.

Exclusion criteria:

History of psychotic features or bipolar disorder.

Substance-induced depression as the primary diagnosis.

Attention deficit hyperactivity disorder (ADHD)

Inclusion criteria:

Diagnosis of ADHD based on DSM-5 criteria.

Exclusion criteria:

Presence of other primary psychiatric disorders (e.g., autism, bipolar disorder).

Substance-induced ADHD as the primary diagnosis.

Bipolar mania

Inclusion criteria:

Diagnosis of bipolar I disorder, current or recent manic episode, as per DSM-5 criteria.

Exclusion criteria:

Presence of other primary psychiatric disorders (e.g., schizophrenia).

Substance-induced mania as the primary diagnosis.

Bipolar depression:

Inclusion criteria:

Diagnosis of bipolar I or bipolar II disorder, current or recent depressive episode, according to DSM-5 criteria.

Exclusion criteria:

Presence of other primary psychiatric disorders (e.g., schizophrenia).

Substance-induced depression as the primary diagnosis.

Methamphetamine-induced psychosis:

Inclusion criteria:

Psychotic symptoms induced by methamphetamine use.

Exclusion criteria:

Presence of pre-existing primary psychiatric disorders (e.g., schizophrenia, bipolar disorder).

Substance-induced psychosis by substances other than methamphetamine.

**2. Genetic datasets for Polygenic risk score(PRS) analyses**

**PGC3-SCZ.** Summary statistics for the largest SCZ GWAS by Psychiatric Genomic Consortium (PGC) were downloaded from the PGC website.^[1]^ To better match with ancestry in PsychENCODE-SCZ and UKB-SCZ datasets, we only included data from the European population (53,386 cases versus 77,258 controls). The summary statistics were for ~6.5M SNPs directly genotyped or imputed with info score > 0.5 (Table S1).

**PsychENCODE-SCZ.** Schizophrenia patients and matched controls were collected through 3 datasets from PsychENCODE Consortium: Common Mind consortium (CMC) [2], Human Brian Collection Core (HBCC),^[2]^ and Lieber Institute for Brain Development (LIBD).^[3]^ We prioritized them as most of these samples have both RNA-seq and DNA array data for analyses of this study. Their phenotype and genotype data, as also described in the original PsychENCODE report,^[4]^ were downloaded from the Synapse portal. In brief, all samples were genotyped using the Illumina platform and imputed against the Haplotype Reference Consortium (HRC) reference panel. To avoid batch effect, we included only one array for each dataset and performed data harmonization before combining them together. Standard quality control steps at sample level (e.g., Caucasian only, unrelated [i.e., removing first-degree relatives], and principal component outlier) and variant level (e.g., minor allele frequency [MAF] > 0.01, Hardy-Weinberg equilibrium [HWE] test *P* > 10^-6^, and imputation info score > 0.5) were adopted. Only biallelic SNPs present in all 3 datasets were retained and combined across all unrelated European (EUR) descent samples. After that, 327 cases and 296 controls with genome-wide genotypes for ~4.4M SNPs were included in this study (Table S1).

**UKB-SCZ**. Eligible schizophrenia patients and non-psychiatric controls were also extracted from the UK Biobank cohort (under application no. 86920).^[5]^ Patients were assessed by self-reported survey (data field 20002 and 20544) or ICD 9 or 10 diagnostic codes (F20 or F25 in data field 41202, 41204, 40001, or 40002), following a recent study with UKB data.^[6]^ All controls were participants completing mental health questionnaire (MHQ), and with the answer “No” to questions for “Unusual and psychotic experiences” (data field 20463, 20468, 20474, and 20471). We further excluded those controls with any major psychiatric disease (depression, anxiety, stress-related disorder, substance misuse, and psychotic disorders) as previously reported.^[7]^ To generate a clean dataset for downstream PRS analysis, we used quality metrics provided by UK Biobank (data field category 100313 that covers genetic ethnic grouping and principal components), and those metrics (e.g., kinship relationship and genotyping quality) generated by KING or PLINK 2.0.^[8]^ At the sample level, we filtered samples by their self-reported ancestry/ethnicity (data field 21000) to include British White; and then applied category 100313 metrics to include European-ancestry individuals without sex mismatch or low genotyping individuals; moreover, we kept only unrelated samples defined by KING. At the variant level, PLINK was used to filter out variants by MAF (0.01), HWE test (1×10^-6^), genotyping rate (0.95), and imputation info score (0.5). This resulted in 869 SCZ patients and 107,860 controls with clean genotypes for ~10M variants (Table S1).

**3. Extraction of characteristic DREGs**

**We used SVA package to correct for batch effects, ensuring that the variability introduced by different batches did not confound our results. To standardize the count matrices of all training datasets for efficiently evaluating relative expression patterns, we used the following formula,**

$$x_{\mathrm{gene}}^{*}=\frac{x_{\mathrm{gene}}-\mu_{\mathrm{gene}}}{\sigma_{\mathrm{gene}}}$$

**where** $x_{\mathrm{gene}}$ **represents the expression value of a specific gene in each sample,** $\mu_{\mathrm{gene}}$ **represents the average expression value of the gene across all samples, and** $\sigma_{\mathrm{gene}}$ **represents the standard deviation of the gene. Recursive feature elimination^[9]^ was employed to identify characteristic DREGs. This method ranks features (genes) based on their importance and iteratively removes less significant features. The final set of remaining features (genes) with the highest accuracy represents the characteristic DREGs. The specific analysis was conducted in the following steps: (1) we chose the support vector machine (SVM) algorithm^[10]^ as our base model due to its exceptional generalization ability and ability to handle complex, noisy, and non-linear data without overfitting**^[11]^**. The average accuracy of the 10-fold cross-validation results from the training set was used as the evaluation metric to determine the number of features (genes) to be retained. (2) We incorporated the trained SVM model into the recursive feature elimination process (n _ features _ to _ select = 1). Each iteration of this process eliminated one feature (gene) at a time. The least important features (genes) were iteratively removed from the current feature set, followed by model training. (3) The importance of each feature (gene) was recorded based on the rankings provided by the recursive feature elimination evaluator. When the average accuracy of the 10-fold cross-validation results from the training set no longer increased with the reduction of features (genes), the remaining features (genes) were considered as the final characteristic DREGs.**

**4. Analysis of the biological basis of DREGs**

**4.1 Integration of protein-protein interaction data from multiple databases**

We conducted an extensive collection and integration of protein-protein interaction (PPI) data to construct a more comprehensive dataset. This dataset consists of information gathered from various databases, including String,^[12]^ Biogrid,^[13]^ Bioplex,^[14]^ CCSB,^[15]^ HINT,^[16]^ HPRD,^[17]^ IntAct,^[18]^ and Mint.^[19]^ Additionally, we incorporated PPI information from a previously published paper^[20]^ into our dataset. To ensure data quality, self-interaction information was excluded, and gene symbols were standardized as the unique identification ID of the molecules to maintain consistency in protein identifiers. After integrating PPI information from different databases, we removed duplicates, resulting in a dataset that includes 24,178 protein-coding genes and 2,544,177 unique PPIs.

**4.2 Analysis of protein-protein interaction networks derived from DREGs**

We utilized the collected interaction data to construct a PPI network and employed Cytoscape^[21]^ to analyze the interaction relationships between proteins encoded by DREGs. Visualizing the PPI network of DREGs allowed for a systematic study of their properties through various network parameters. These parameters include node degree, closeness centrality, betweenness centrality, clustering coefficient, and average shortest path length. Node degree signifies the number of connections a node has with other nodes, helping identify proteins with more interactions and potential key roles in biological processes. Closeness centrality measures how close a node is to others in the network, indicating its ability to spread information efficiently. Betweenness centrality assesses a node's bridging capability between different network modules. Nodes with high betweenness centrality often serve as crucial connectors. The clustering coefficient reflects the interconnectedness of adjacent nodes, representing the connectivity and tightness of local areas in the network. Lastly, the average shortest path length indicates how quickly information spreads in the network. The above-mentioned network parameters were compared between DREGs and the background genes (BG genes) consisting of 19,406 human protein-coding genes from GENECODE annotation (version 44) using the Wilcox rank sum test in R.

**4.3 Analysis of hub genes and densely connected modules**

Hub genes were defined as DREGs that directly interact with at least 20 other DREGs. To identify densely connected modules, we employed clusterONE,**^[22]^** an integrated plug-in in Cytoscape specifically designed for PPI network module detection. clusterONE helps identify functional modules or subnetworks within the PPI network. In this analysis, we used the following parameter settings: Minimum size = 3, Minimum density = Auto, Edge weight = unweighted, Node penalty = 2, Haircut threshold = 0, Merging method = Single-pass, Similarity = Match coefficient, Overlap threshold = 0.8, seeding method = From unused nodes. Modules that were statistically significant (below the significance test threshold of 0.05) and larger than the minimum size were considered.

**4.4 Pathway enrichment analysis**

To gain insights into the potential biological functions of DREGs, hub genes, and densely connected modules, we performed GO (Gene Ontology) and KEGG pathway enrichment analyses. These analyses were conducted separately for genes within DREGs, hub genes, and each functional module. Pathways with a *P*-value < 0.05 were considered statistically significant, providing valuable information about the functional roles of the analyzed gene sets.

**4.5 Analysis of expression profiles of DREGs in brain transcriptomic databases**

To investigate the expression patterns of DREGs in various human brain tissues, we obtained expression data (GTEx Analysis V8) from the GTEx database (http://gtexportal.org/). This database offers RNA sequencing-based expression values represented as Transcripts Per Kilobase (TPM). We extracted the expression values of genes within each gene set (DREGs, densely connected modules, hub genes, BG genes) and calculated the median expression value for each specific brain tissue type, allowing for determination of the expression levels of the gene sets in different brain tissues.

For brain developmental expression analysis, we obtained RNA sequencing-based expression data from the BrainSpan database (http://www.brainspan.org/). This database provides gene expression values (Reads Per Kilobase of transcript per Million mapped reads, or RPKM) at both prenatal and postnatal developmental stages of brain. We extracted the expression values of genes within each gene set and calculated the median expression value for each developmental stage.

To analyze expression profiles in different brain regions, we obtained gene expression profiles from the HBT database (https://hbatlas.org/). For each brain region, we extracted the expression values of genes within each gene set and calculated the median expression value to represent the expression level of the gene set in that specific brain region.

Lastly, we focused on the expression analysis of cell types in the middle temporal gyrus (MTG) and anterior cingulate gyrus (CgGr) brain regions, which are related to SCZ[23]. Expression data of different cell types within these brain regions were obtained from the Allen Brain Atlas (https://portal.brain-map.org/) database. We extracted the expression values of genes within each gene set and calculated the median expression value for each cell type to represent the expression level of the gene set in that specific cell type.

The above analyses allowed us to assess the expression patterns of DREGs in different tissues, developmental stages, brain regions, and cell types of the human brain, providing valuable insights into the functional roles and expression patterns of DREGs in the context of SCZ.

1. **Preparation of animal models and qRT-PCR validation of key DREGs**

We identified 19 key DREGs, including 8 hub genes, 8 genes in module1, and 3 genes in module2, with 10 of them (*ADORA2A*,^[24]^ *ENTPD1*,^[25]^ *PLXNA2*,^[26]^ *SEMA7A*, ^[27]^ *ESR1*,^[28]^ *GRB2*,^[29]^ *STAT3*,^[30]^ *BRD4*,^[31]^ *TRIM28*,^[32]^ *MYH9*^[33]^) previously linked to SCZ. To validate their expression patterns, we focused on 9 novel genes (*BICD1, IFFO1, ARFGAP1, KDELR3, CYTH2, PACSIN2, PLXND1, CDK9, DOT1L*) using an SCZ animal model induced by MK-801.

**5.1 Animals**

Male C57BL/6 J mice (n=8 for each group) were obtained from Beijing Vital River Laboratory Animal Technology Co., Ltd. (Beijing, China). Upon arrival, the mice were housed in clean environmental standard group cages with a maximum of 4 animals per cage. They were acclimated for 1 week under a 12:12 h light/dark cycle with ad libitum access to food and water. All experimental procedures were approved by the Institutional Animal Care and Use Committee of Xi'an Jiaotong University.

**5.2 MK-801 treatment**

To model SCZ-related phenotypes in animals, we utilized the NMDA receptor antagonist MK-801 (MedChem Express, Shanghai, China).^[34]^ The experimental mice were randomly assigned to either the MK-801 group or the control group. MK-801 was dissolved in saline to a concentration of 0.1 mg/ml immediately before administration. The mice received intraperitoneal injections of MK-801 or saline once a day for 21 consecutive days.^[34]^ After the final injection, the mice were euthanized.

**5.3 Behavioral testing**

**Open field test**

To assess positive symptoms of SCZ, an open field test (OFT) was conducted following the final administration of MK-801, as previously described. Individual mice were placed in metal test chambers measuring 43 × 43 × 43 cm, and their movements were tracked using the Smart 3.0 video tracking system. Over a 10-minute period, the total distance traveled throughout the test area and the time spent in the central zone (21.50 × 21.50 cm) were recorded.^[34]^

**Y-Maze Test**

The Y-Maze test utilized a Y-shaped apparatus with three arms of equal size (30 cm long, 6 cm wide with walls, and 15 cm high), namely the start arm, familiar arm, and novel arm.^[35]^ The experiment involved two phases: during the habituation phase, the novel arm was covered for the first 10 minutes while the other arms remained accessible. Subsequently, in the test phase, which occurred 1 hour after training, the mice were given 5 minutes to freely explore all three arms of the maze. The Smart 3.0 software recorded the test phase for analysis.

**Elevated plus maze**

The elevated plus maze (EPM) consisted of a plus-shaped platform with two open arms (33 × 6 cm) and two closed arms, all extending from a 6 × 6 cm central area. The platform was elevated 50 cm above the ground. During the 5-minute trial, the time spent in the open and closed arms was measured using Smart 3.0 software. This assessment served as an indicator of anxiety-like behavior and positive symptoms of SCZ.^[34]^

**Forced swimming test**

The forced swimming test (FST) was conducted to evaluate symptoms of SCZ.^[36]^ Each mouse was placed in a 10-liter transparent plastic cylinder filled with tap water at a consistent temperature of 25 ± 1°C and a depth of 30 cm. The mouse was allowed to move freely in the water for 6 minutes, and immobility, defined as the absence of movements except those necessary to prevent drowning, was recorded during the last 5 minutes of the test. Smart 3.0 software facilitated the analysis of immobility duration.

**Prepulse inhibition (PPI) test**

The PPI experiment was conducted in a standard sound-attenuated cabinet that was calibrated before testing. It was carried out in two phases over two consecutive days. The first phase was the acclimation phase, during which mice were acclimated to a Plexiglas cylinder with a background noise of approximately 65 dB (white noise) for 5 minutes. In the second phase, the PPI was evaluated. During this phase, mice were exposed to six blocks of seven trial types presented in a pseudorandom order. The average inter-trial interval was 15 seconds. The trial types included: In trial 1, basal startle responses were measured using a 40-ms 120 dB startle-only pulse. Trials 2-6 involved prepulse tests, where three paired-pulse stimuli were applied in random order. Each prepulse consisted of a 20-ms acoustic stimulus of 70, 75, or 80 dB, followed by a 120 dB startle stimulus 100 ms later. Trial 7 was a 120 dB startle-only trial with 5 pulses and a 15-second interval. The experiment consisted of seven trials. The PPI scores were calculated. PPI scores were calculated as a percentage using the formula: 100% * (1 - startle response for pulse with pre) / startle response for pulse alone.

**5.4 Sample preparation, RNA extraction, and reverse transcription**

Blood was extracted from the orbital veins of the mice under anesthesia at 24 hours following the last MK-801 injection. The blood samples were then centrifuged at 2000 rpm for 15 minutes at 4°C to obtain the plasma. The plasma samples were promptly stored at -80°C. Furthermore, the brains of the mice were also obtained, and the prefrontal cortex (PFC) was swiftly dissected and flash-frozen in liquid nitrogen. Total RNA was extracted from the mouse PFC and plasma using the DNA/RNA/protein kit (Omega, USA). The NanoDrop spectrophotometer (Thermo Scientific, USA) was used to determine the concentration and quality of the extracted RNA. 500 ng of total RNA was reverse transcribed into 10 μl of cDNA using the PrimeScriptTM RT Master Mix (TaKaRa, Japan) with the following parameters: 37°C for 15 minutes, 85°C for 5 seconds, and 4°C for 5 minutes.

**5.5 qRT-PCR**

Quantitative reverse transcription-polymerase chain reaction (qRT-PCR) was performed using the SYBR Premix Ex Taq II kit (TaKaRa, Japan) in a Bio-Rad CFX96 detection instrument (Bio-Rad, USA). The PCR conditions consisted of an initial denaturation step at 95°C for 30 seconds, followed by 40 cycles of denaturation at 95°C for 5 seconds and annealing/extension at 60°C for 30 seconds. The housekeeping gene Gapdh was used as an internal control for qRT-PCR, and the relative expression levels were determined using the 2-^ΔΔCt^ method. The primer sequences are provided in Table S2. An independent-samples t-test was performed to compare the expression differences in the 9 key DREGs between the control group and the MK-801 group. The data were presented as means ± standard error of the mean (SEM), with *P*-values <0.05 considered statistically significant.

**6. Hyperparameter tuning of candidate models**

We optimized hyperparameters of these basic learners with a Tree Parzen Estimator (TPE)-based Bayesian optimization method based on a 10-fold cross-validation (CV). The object functions of these 8 models are (eq1 to eq9).

Decision Tree object function:

$$Gini(D) = \sum_{i=1}^{n} p(x_{i})*(1-p(x_{i})) (1)$$

Where $p(x_{i})$ is the probability of occurrence of class $x_{i}$, and n is the number of classes. Gini(D) reflects the probability that two samples taken at random from dataset D have inconsistent category labels. Therefore, the smaller Gini(D) is, the higher the purity of the dataset D.

Extra Tree object function:

$${Score}_{C}(s,S) = \frac{2I_{C}^{s}(S)}{H_{s}(S)+H_{C}(S)} (2)$$

For a sample S and a split s, the measure of information gain is given by the above equation. Where $H_{C}(S)$ is the entropy of the classification in S, $H_{s}(S)$ is the split entropy, and $I_{C}^{s}(S)$ is the mutual information of the split outcome and classfication.

GBDT object function:

$$H_{t}\left( x_{i} \right)=H_{t-1}\left( x_{i} \right)+\eta f_{t}\left( x_{i} \right) (3)$$

$$r_{it}=-\frac{\partial l\left( y_{i},H_{t-1}\left( x_{i} \right) \right)}{\partial H_{t-1}\left( x_{i} \right)} (4)$$

$H_{t}\left( x_{i} \right)$is the prediction result of sample number at the *t`th* iteration on the ensemble machine learning algorithm.$\eta$ was the learning rate and $f_{t}\left( x_{i} \right)$ was the result of a weak estimator fit. The labels that $f_{t}\left( x_{i} \right)$ need to fit when training was the $r_{it} ($pseudo-residuals) of the sample(*x_i_*). The loss in our GBDT model was cross entropy loss and the criterion of the weak estimator was squared error.

Logistic Regression object function:

$${L(\omega)}_{logist}=\sum_{i=1}^{N} \left[ y_{i}\left( \omega*x_{i} \right)-\log\left( 1+\exp\left( \omega*x_{i} \right) \right) \right] (5)$$

($x_{i},y_{i}$) is the sample of the dataset and $\omega$ is the parameter. ${L(\omega)}_{logist}$ is the Log-likelihood function.

MLP object function:

$$E = \lambda\frac{1}{m}\sum_{k=1}^{m} E_{k}+(1-\lambda)\sum_{i} \omega_{i}^{2} (6)$$

Where $E_{k}$represents the error on the $k^{th}$training example, $\omega_{i}$ represents the connection weight. $\lambda$ is use for the compromise between empirical error and network complexity.

Random forest object function:

$$h_{t}=\pi\left( D,D_{bs} \right) (5)$$

$$H\left( x_{i} \right)=argmax\sum_{t=1}^{T} I\left( h_{t}\left( x_{i} \right)=y_{i} \right) y_{i}\in Y (7)$$

($x_{i},y_{i}$) is the sample of the dataset ($D_{bs}$), $h_{t}$ is the function of the decision tree in a random forest, and $I$ is the instruction function.

SVM object function:

$${Obj}_{svm}={max}_{\alpha}{min}_{\omega,b}\left( \frac{1}{2}\left| \left| \omega\right| \right|^{2}-\sum_{i=1}^{N} \alpha_{i}y_{i}\left( \omega x_{i}+b \right)+\sum_{i=1}^{N} \alpha_{i} \right) (8)$$

In the object function of support-vector networks, $\omega$ and $b$ are the hyperplane parameters. $\alpha_{i}$ is the Lagrange multiplier.

XGboost object function:

$${Obj}_{xgb}=\sum_{i=1}^{m} \left[ f_{t}\left( x_{i} \right)g_{i}+\frac{1}{2}\left( f_{t}\left( x_{i} \right) \right)^{2}h_{i} \right]+\gamma T+\frac{1}{2}\alpha\sum_{j=1}^{T} \left| \omega_{j} \right|+\frac{1}{2}\lambda\sum_{j=1}^{T} \omega_{j}^{2} (9)$$

In this objective function, $g_{i}$ and $h_{i}$were the first and second derivatives, respectively. T was identified as the number of leaf nodes. Meanwhile, L1 regularization and L2 regularization were utilized to control the complexity of the model. $f_{t}\left( x_{i} \right)$and $\omega_{j}$ are both predictors of model output (leaf node output). Numerically $f_{t}\left( x_{i} \right)$=$\omega_{j}$, for any sample i on leaf *j*.

**Supplementary Tables**

**Table S1 Results of differential expression analysis of four training sets**

Results were provided in another Excel file for better presentation.

**Table S2 Pathway enrichment analysis results of differentially expressed genes in four training sets**

Results were provided in another Excel file for better presentation.

**Table S3 Results of significant pathways shared by the four training sets and the significantly expressed genes involved**

Results were provided in another Excel file for better presentation.

**Table S4 Summary of 184 disease-responsive essential genes**

| 184 disease-responsive essential genes of SCZ | | | | | |
| --- | --- | --- | --- | --- | --- |
| KDELR3 | ACTR1A | PRKRA | BBS2 | CEP152 | RING1 |
| DNAJB1 | SF3B5 | MYB | PACSIN2 | ZYX | DLG2 |
| HSPB1 | DSG2 | DENND3 | RAB11FIP1 | STMN1 | CAMK4 |
| VPS37C | ADORA2A | EXD2 | AMN1 | PALLD | PRPF40A |
| STK3 | CCNI | TAGAP | FGF13 | ULK1 | SNX24 |
| SMARCE1 | SYT11 | PTPN6 | ARPC5 | PHF1 | NPHP1 |
| SLC30A7 | RNF165 | DDX54 | BICD1 | FOXP1 | CD47 |
| DBN1 | UFM1 | TRIP6 | RRM2B | STK35 | ARID2 |
| KSR1 | EML6 | LRWD1 | LRRC8C | SEMA7A | SOCS2 |
| PEX5 | SVIP | STON2 | NCLN | MAP3K14 | CELF2 |
| SHROOM3 | GALK1 | UNC45A | BCL2 | MAPK7 | DOT1L |
| MAP2K3 | TNKS1BP1 | KRT5 | CYTH2 | FASN | ARHGAP27 |
| PSTPIP1 | GNL3L | ARL2 | SMURF1 | SIRT5 | SEPTIN1 |
| RPL29 | CASC3 | RUFY3 | GRK2 | SLIT1 | MAP3K13 |
| PDCD2 | MYH9 | TNS3 | CPNE9 | RNF168 | ESCO1 |
| ITPKB | TICAM1 | CSNK1G2 | RLF | SUPT6H | PACS1 |
| TRPC1 | TRPM2 | AGPAT5 | APH1A | WAPL | MPZL1 |
| NEDD4 | ADRM1 | GSR | TOM1 | ESR1 | SPTBN1 |
| MCF2L2 | PPARGC1A | GRB2 | TSNARE1 | SHPRH | AP1AR |
| FBXO25 | KCTD17 | TOMM40L | CDK9 | KIF16B | TRPC4AP |
| PSME3 | TADA1 | KATNAL2 | MYBBP1A | IFFO1 | MAPK8 |
| ARHGAP24 | UBE3C | TRIM28 | NISCH | TRAF3IP2 | MED28 |
| BRD4 | ADAP1 | ARFGAP1 | CTNNBIP1 | STAT3 | PLXNA2 |
| HSBP1L1 | PRDM2 | UBE3D | ZNF451 | ARPC3 | GET4 |
| RHNO1 | BCL6 | TBCA | ERCC6L2 | RIOK1 | CSK |
| LIMK2 | FGD3 | ATAD1 | CRTC1 | PTP4A3 | FEZ1 |
| CORO7 | RAB3D | MTMR9 | BAIAP3 | DENND2B | PLXND1 |
| PFN2 | TRIP11 | EML5 | DNM1L | NUDT1 | CDT1 |
| RNF166 | TNIP1 | UTRN | RABGGTB | MYO1B | PHF23 |
| ZCCHC4 | CENPC | ENTPD1 | TOMM5 | ZSWIM4 | FAF1 |
| PCDHGB1 | TET1 | SYNGAP1 | PRKACA |  |  |

**Table S5 Summary of the constructed interactome dataset**

| Datasets | PPI number (after remove self links and replications) |
| --- | --- |
| STRING | 1,477,610 |
| Biogrid | 1,065,411 |
| Bioplex | 186,464 |
| CCSB | 52,523 |
| MINT | 49,666 |
| HINT | 114,654 |
| HPRD | 37,080 |
| IntAct | 522,352 |
| Retrieved from a literature* | 136,614 |
| Merged (remove duplicate records in 9 datasets): 2,544,177 | |

*Menche, J., et al., Disease networks. Uncovering disease-disease relationships through the incomplete interactome. Science, 2015. 347(6224): p. 1257601.

**Table S6 Results of GO and KEGG pathway enrichment analysis of 184 disease-responsive essential genes**

Results were provided in another Excel file for better presentation.

**Table S7 Results of GO and KEGG pathway enrichment analysis of 8 hub genes**

Results were provided in another Excel file for better presentation.

**Table S8 Results of GO and KEGG pathway enrichment analysis of module 1**

Results were provided in another Excel file for better presentation.

**Table S9 Results of GO and KEGG pathway enrichment analysis of module 2**

Results were provided in another Excel file for better presentation.

**Table S10 The top20 most repeated significant GO and KEGG pathways in Top32 disease-responsive essential genes**

Results were provided in another Excel file for better presentation.

**Table S11 The top20 most repeated significant GO and KEGG pathways in hub genes**

Results were provided in another Excel file for better presentation.

**Table S12 The top20 most repeated significant GO and KEGG pathways in module 1**

Results were provided in another Excel file for better presentation.

**Table S13 The top20 most repeated significant GO and KEGG pathways in module 2**

Results were provided in another Excel file for better presentation.

**Table S14 Polygenic risk for schizophrenia associated with genome-wide PRS and DREG PRS in PGC3-UKB and PGC3-PsychENOCDE dataset**

| \| Train dataset \| Target dataset \| Variant set \| SNP *P*-threshold \| Number  of SNPs \| OR \| 95%CI \| *P* ^*^ \| *P*_perm ^#^ \| \| --- \| --- \| --- \| --- \| --- \| --- \| --- \| --- \| --- \| \| PGC3-SCZ  (53,386 vs 77,258) \| UKB-SCZ  (869 vs 107,860) \| whole genome \| 0.2 \| 41038 \| 2.15 \| 2.01-2.31 \| 1.59x10^-104^ \| <1.00x10^-5^ \| \| 184 DREGs \| 1.0x10^-6^ \| 14 \| 1.19 \| 1.12-1.28 \| 2.47x10^-07^ \| <1.00x10^-5^ \| \| PsychENCODE-SCZ  (327 vs 296) \| whole genome \| 0.4 \| 85256 \| 8.40 \| 5.58-12.64 \| 1.70x10^-24^ \| <1.00x10^-5^ \| \| 184 DREGs \| 0.5 \| 977 \| 1.34 \| 1.14-1.58 \| 4.13x10^-04^ \| 3.01x10^-3^ \|   ^*^ *P*-value is for PRS-disease association by logistic regression with adjustment of age, gender, and top PCs. |
| --- | --- | --- | --- | --- | --- | --- | --- | --- | --- | --- | --- | --- | --- | --- | --- | --- | --- | --- | --- | --- | --- | --- | --- | --- | --- | --- | --- | --- | --- | --- | --- | --- | --- | --- | --- | --- | --- | --- | --- | --- |
| ^#^ Permutation is based on permuting sample label for 100,000 times. |
| OR stands for odds ratio, 95%CI for 95% confidence interval, DREG for disease-responsive essential gene. |

**Table S15 Specific parameters for model optimization**

| Models | Parameters |
| --- | --- |
| Decision Tree | 'ccp_alpha': 0.0, 'class_weight': None, 'criterion': 'gini', 'max_depth': 88, 'max_features': 170, 'max_leaf_nodes': None, 'min_impurity_decrease': 0.0, 'min_samples_leaf': 38, 'min_samples_split': 58, 'min_weight_fraction_leaf': 0.0, 'random_state': 30, 'splitter': 'best' |
| Extra Tree | 'bootstrap': True, 'ccp_alpha': 0.0, 'class_weight': None, 'criterion': 'entropy', 'max_depth': 49, 'max_features': 165, 'max_leaf_nodes': None, 'max_samples': 0.84, 'min_impurity_decrease': 0.002, 'min_samples_leaf': 2, 'min_samples_split': 2, 'min_weight_fraction_leaf': 0.0, 'n_estimators': 245, 'n_jobs': None, 'oob_score': False, 'random_state': 30, 'verbose': False, 'warm_start': False |
| Gradient Tree Boosting | 'ccp_alpha': 0.0, 'criterion': 'friedman_mse', 'init': None, 'learning_rate': 0.046, 'loss': 'log_loss', 'max_depth': 6, 'max_features': 24, 'max_leaf_nodes': None, 'min_impurity_decrease': 0.4, 'min_samples_leaf': 9, 'min_samples_split': 9, 'min_weight_fraction_leaf': 0.0, 'n_estimators': 135, 'n_iter_no_change': None, 'random_state': 30, 'subsample': 0.41300000000000003, 'tol': 0.0001, 'validation_fraction': 0.1, 'verbose': False, 'warm_start': False |
| Logistic Regression | 'C': 1.9000000000000001, 'class_weight': None, 'dual': False, 'fit_intercept': True, 'intercept_scaling': 1, 'l1_ratio': None, 'max_iter': 100, 'multi_class': 'auto', 'n_jobs': None, 'penalty': 'l2', 'random_state': 30, 'solver': 'lbfgs', 'tol': 0.0001, 'verbose': 0, 'warm_start': False |
| Multi-layer Perceptron | 'activation': 'logistic', 'alpha': 5e-05, 'batch_size': 'auto', 'beta_1': 0.9, 'beta_2': 0.999, 'early_stopping': False, 'epsilon': 1e-08, 'hidden_layer_sizes': (100,), 'learning_rate': 'constant', 'learning_rate_init': 0.001, 'max_fun': 15000, 'max_iter': 430, 'momentum': 0.9, 'n_iter_no_change': 10, 'nesterovs_momentum': True, 'power_t': 0.5, 'random_state': 30, 'shuffle': True, 'solver': 'adam', 'tol': 0.0001, 'validation_fraction': 0.1, 'verbose': False, 'warm_start': False |
| Random Forest | 'bootstrap': True, 'ccp_alpha': 0.0, 'class_weight': None, 'criterion': 'entropy', 'max_depth': 18, 'max_features': 83, 'max_leaf_nodes': None, 'max_samples': 769, 'min_impurity_decrease': 0.001, 'min_samples_leaf': 6, 'min_samples_split': 9, 'min_weight_fraction_leaf': 0.0, 'n_estimators': 129, 'n_jobs': None, 'oob_score': False, 'random_state': 30, 'verbose': False, 'warm_start': False |
| SVM | 'C': 1.51, 'break_ties': False, 'cache_size': 200, 'class_weight': None, 'coef0': 0.0, 'decision_function_shape': 'ovr', 'degree': 3, 'gamma': 0.73, 'kernel': 'linear', 'max_iter': -1, 'probability': True, 'random_state': 30, 'shrinking': True, 'tol': 0.001, 'verbose': False |
| XGboost | 'objective': 'binary:logistic', 'use_label_encoder': None, 'base_score': None, 'booster': 'gblinear', 'callbacks': None, 'colsample_bylevel': None, 'colsample_bynode': 0.58, 'colsample_bytree': 0.51, 'early_stopping_rounds': None, 'enable_categorical': False, 'eval_metric': None, 'feature_types': None, 'gamma': 1700000.0, 'gpu_id': None, 'grow_policy': None, 'importance_type': None, 'interaction_constraints': None, 'learning_rate': None, 'max_bin': None, 'max_cat_threshold': None, 'max_cat_to_onehot': None, 'max_delta_step': None, 'max_depth': 18, 'max_leaves': None, 'min_child_weight': None, 'missing': nan, 'monotone_constraints': None, 'n_estimators': 150, 'n_jobs': None, 'num_parallel_tree': None, 'predictor': None, 'random_state': 30, 'reg_alpha': None, 'reg_lambda': 0.0, 'sampling_method': None, 'scale_pos_weight': None, 'subsample': 0.96, 'tree_method': None, 'validate_parameters': None, 'verbosity': None, 'verbose': False |

**Table S16. Data resources and tailored analysis included in this study**

| **Aim** | **Data Type** | **Cohort Name** | **Case vs Control** | **Analysis^#^** |
| --- | --- | --- | --- | --- |
| Discovery | RNA-seq | CMC | 264 vs 294 | DREGs identification, training and  cross validation for characterization modeling |
|  |  | LIBD | 175 vs 318 |  |
|  |  | HBCC | 97 vs 220 |  |
| Annotation^*^ | SNP array | PsychENCODE  (CMC/HBCC/LIBD) | 327 vs 296 | PRS target data |
|  |  | UKB | 869 vs 107,860 |  |
|  | GWAS summary statistics | PGC3 | 53,386 vs 77,258 | PRS training data |

^*^ To avoid heterogeneity, only GWAS data from European-ancestry samples were used in genomic annotation. Thus, the included sample of PsychENCODE (CMC/HBCC/LIBD) is smaller than the original sample.

^#^ PRS is for polygenic risk scoring. In PRS, PGC3 data was used as the training cohort, and PsychENCODE (CMC/HBCC/LIBD) or UKB data as the target cohort.

**Table S17 Summary of primers for quantitative PCR detection of 9 key DREGs**

| Gene | Forward primer sequence (5′-3′) | Reverse primer sequence (5′-3′) |
| --- | --- | --- |
| BICD1 | TGTCCACATTGAAGCAGAACCA | GGCTTCTTCCAACTGATGCT |
| IFFO1 | GCCAAGAACGACATGAACCG | CGCAGTGAAAGCAGGAGACT |
| ARFGAP1 | GAACACAGTGCCACCTCAGA | TGTAGCACCCTCCTTTGCTG |
| KDELR3 | GCCAACTGGATCAGGCGGTA | GCACCGGGAGGCTTAATTTC |
| CYTH2 | ACAGCTCATCAAAGCCTGCAA | CACACTTACAGCAGCCTGGAT |
| PACSIN2 | GGACACGACCTCAGGAACAG | TCTAAACGTCCCTTGCACCA |
| PLXND1 | TGCCCTCGGAGATTGATGTG | ATCATAGGCAGGGCCAGGTA |
| CDK9 | ATGCAGGGTAACACAGAGCAG | CGCTTCTGGCCCTTCACAAG |
| DOT1L | CAGCAGTGCCCGAATTGAGA | TTGATAGGCTCGCTCGAAGG |
| Gapdh | TGTGTCCGTCGTGGATCTGA | TTGCTGTTGAAGTCGCAGGAG |

**Supplementary Figures**


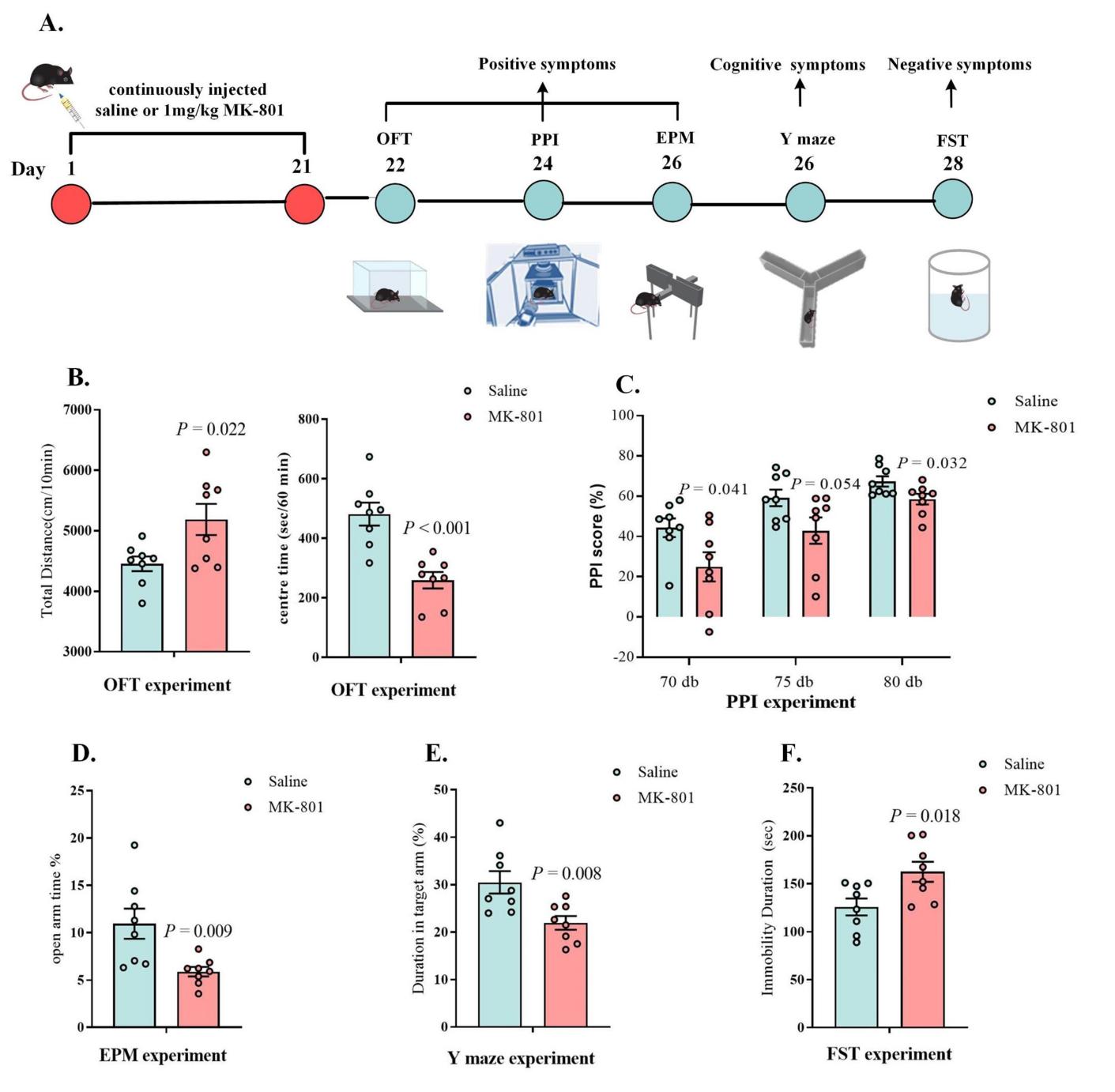


**Figure S1 Behavioral effects of repeated MK-801 Treatment on mice in schizophrenia-relevant tests**

(A) The timeline for assessing SCZ-relevant behavioral tests in mice treated with MK-801 . The behavioral tests conducted include the open field test (OFT), pre-pulse inhibition (PPI), elevated plus maze (EPM), Y maze test, and forced swimming test (FST). (B) The effects of repeated MK-801 treatment on locomotor distance and center time in the OFT of C57BL/6 mice.(C) The effects of repeated MK-801 treatment on PPI scores in the PPI experiment.(D) Comparison of the proportions of time spent in open arms in the EPM test between MK-801 treated mice and control mice.(E) The effects of repeated MK-801 treatment on the percentage of time spent in novel arms in the Y maze test. (F) The effects of repeated MK-801 treatment on immobility time in the FST. Statistical comparisons were performed using Student's t-test. Data are presented as means ± SEM (n=8 per group). Significant differences between SCZ and control groups are indicated in the figure (*P* < 0.05).

**
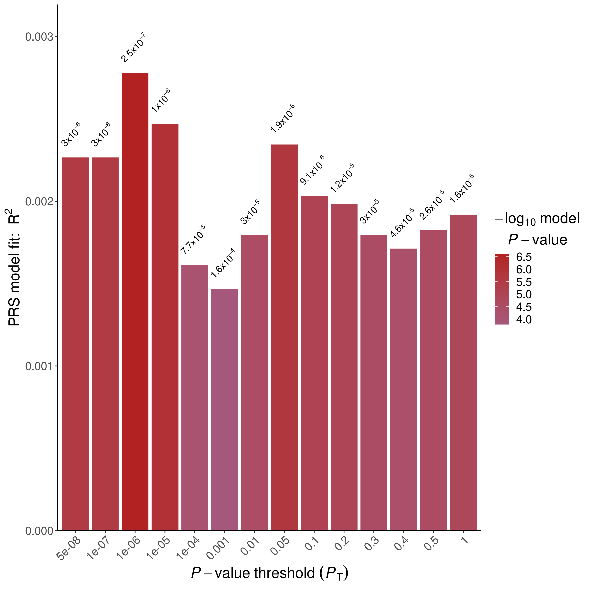

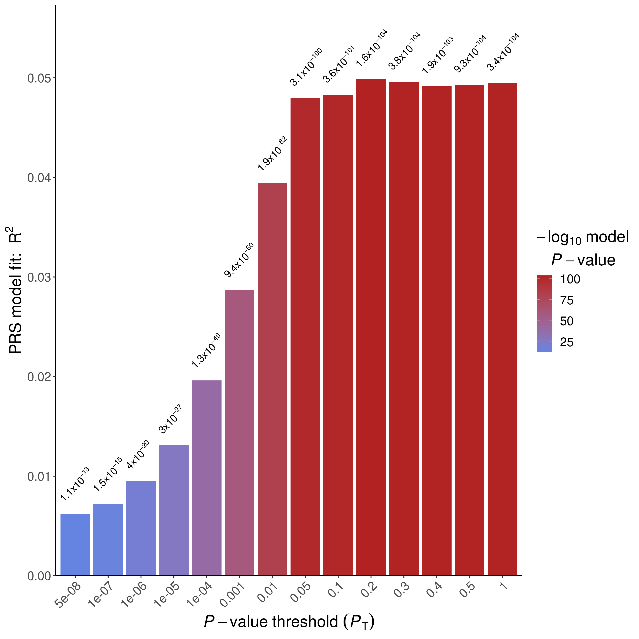
A B**

**C D**

**
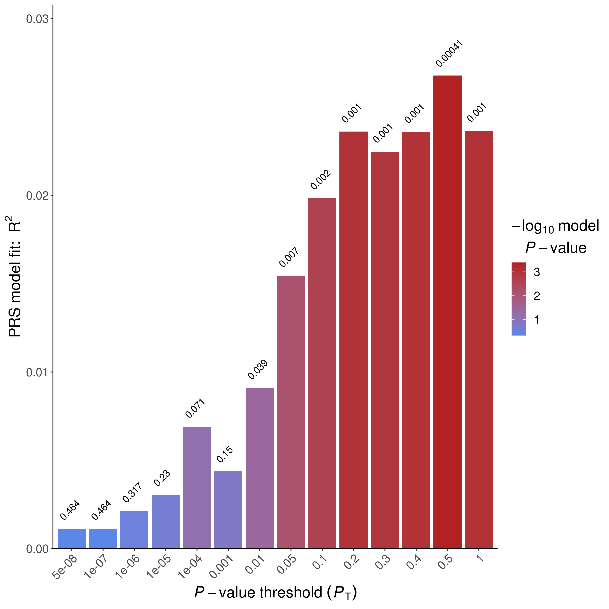

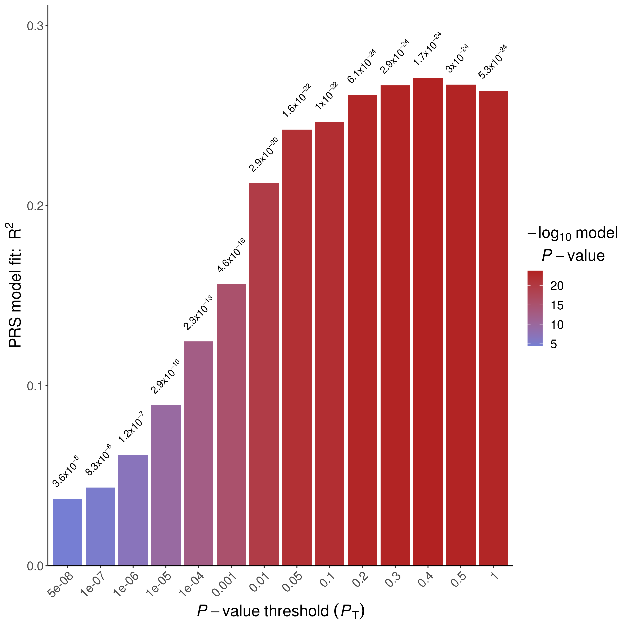
**

**Figure S2. Bar plots for SCZ PRS regression in UKB and PsychENOCDE cohorts**

A and B (upper left and right): PGC3-SCZ PRS fitting on UKB using genome-wide SNPs or SNPs from 184 DREGs only. C and D (lower left and right): PGC3-SCZ PRS fitting on PsychENCODE using genome-wide SNPs or SNPs from 184 DREGs only.

**References**

[1] V. Trubetskoy, A. F. Pardiñas, T. Qi, G. Panagiotaropoulou, S. Awasthi, T. B. Bigdeli, J. Bryois, C. Y. Chen, C. A. Dennison, L. S. Hall, M. Lam, K. Watanabe, O. Frei, T. Ge, J. C. Harwood, F. Koopmans, S. Magnusson, A. L. Richards, J. Sidorenko, Y. Wu, J. Zeng, J. Grove, M. Kim, Z. Li, G. Voloudakis, W. Zhang, M. Adams, I. Agartz, E. G. Atkinson, E. Agerbo, M. Al Eissa, M. Albus, M. Alexander, B. Z. Alizadeh, K. Alptekin, T. D. Als, F. Amin, V. Arolt, M. Arrojo, L. Athanasiu, M. H. Azevedo, S. A. Bacanu, N. J. Bass, M. Begemann, R. A. Belliveau, J. Bene, B. Benyamin, S. E. Bergen, G. Blasi, J. Bobes, S. Bonassi, A. Braun, R. A. Bressan, E. J. Bromet, R. Bruggeman, P. F. Buckley, R. L. Buckner, J. Bybjerg-Grauholm, W. Cahn, M. J. Cairns, M. E. Calkins, V. J. Carr, D. Castle, S. V. Catts, K. D. Chambert, R. C. K. Chan, B. Chaumette, W. Cheng, E. F. C. Cheung, S. A. Chong, D. Cohen, A. Consoli, Q. Cordeiro, J. Costas, C. Curtis, M. Davidson, K. L. Davis, L. de Haan, F. Degenhardt, L. E. DeLisi, D. Demontis, F. Dickerson, D. Dikeos, T. Dinan, S. Djurovic, J. Duan, G. Ducci, F. Dudbridge, J. G. Eriksson, L. Fañanás, S. V. Faraone, A. Fiorentino, A. Forstner, J. Frank, N. B. Freimer, M. Fromer, A. Frustaci, A. Gadelha, G. Genovese, E. S. Gershon, M. Giannitelli, I. Giegling, P. Giusti-Rodríguez, S. Godard, J. I. Goldstein, J. González Peñas, A. González-Pinto, S. Gopal, J. Gratten, M. F. Green, T. A. Greenwood, O. Guillin, S. Gülöksüz, R. E. Gur, R. C. Gur, B. Gutiérrez, E. Hahn, H. Hakonarson, V. Haroutunian, A. M. Hartmann, C. Harvey, C. Hayward, F. A. Henskens, S. Herms, P. Hoffmann, D. P. Howrigan, M. Ikeda, C. Iyegbe, I. Joa, A. Julià, A. K. Kähler, T. Kam-Thong, Y. Kamatani, S. Karachanak-Yankova, O. Kebir, M. C. Keller, B. J. Kelly, A. Khrunin, S. W. Kim, J. Klovins, N. Kondratiev, B. Konte, J. Kraft, M. Kubo, V. Kučinskas, Z. A. Kučinskiene, A. Kusumawardhani, H. Kuzelova-Ptackova, S. Landi, L. C. Lazzeroni, P. H. Lee, S. E. Legge, D. S. Lehrer, R. Lencer, B. Lerer, M. Li, J. Lieberman, G. A. Light, S. Limborska, C. M. Liu, J. Lönnqvist, C. M. Loughland, J. Lubinski, J. J. Luykx, A. Lynham, M. Macek, Jr., A. Mackinnon, P. K. E. Magnusson, B. S. Maher, W. Maier, D. Malaspina, J. Mallet, S. R. Marder, S. Marsal, A. R. Martin, L. Martorell, M. Mattheisen, R. W. McCarley, C. McDonald, J. J. McGrath, H. Medeiros, S. Meier, B. Melegh, I. Melle, R. I. Mesholam-Gately, A. Metspalu, P. T. Michie, L. Milani, V. Milanova, M. Mitjans, E. Molden, E. Molina, M. D. Molto, V. Mondelli, C. Moreno, C. P. Morley, G. Muntané, K. C. Murphy, I. Myin-Germeys, I. Nenadić, G. Nestadt, L. Nikitina-Zake, C. Noto, K. H. Nuechterlein, N. L. O'Brien, F. A. O'Neill, S. Y. Oh, A. Olincy, V. K. Ota, C. Pantelis, G. N. Papadimitriou, M. Parellada, T. Paunio, R. Pellegrino, S. Periyasamy, D. O. Perkins, B. Pfuhlmann, O. Pietiläinen, J. Pimm, D. Porteous, J. Powell, D. Quattrone, D. Quested, A. D. Radant, A. Rampino, M. H. Rapaport, A. Rautanen, A. Reichenberg, C. Roe, J. L. Roffman, J. Roth, M. Rothermundt, B. P. F. Rutten, S. Saker-Delye, V. Salomaa, J. Sanjuan, M. L. Santoro, A. Savitz, U. Schall, R. J. Scott, L. J. Seidman, S. I. Sharp, J. Shi, L. J. Siever, E. Sigurdsson, K. Sim, N. Skarabis, P. Slominsky, H. C. So, J. L. Sobell, E. Söderman, H. J. Stain, N. E. Steen, A. A. Steixner-Kumar, E. Stögmann, W. S. Stone, R. E. Straub, F. Streit, E. Strengman, T. S. Stroup, M. Subramaniam, C. A. Sugar, J. Suvisaari, D. M. Svrakic, N. R. Swerdlow, J. P. Szatkiewicz, T. M. T. Ta, A. Takahashi, C. Terao, F. Thibaut, D. Toncheva, P. A. Tooney, S. Torretta, S. Tosato, G. B. Tura, B. I. Turetsky, A. Üçok, A. Vaaler, T. van Amelsvoort, R. van Winkel, J. Veijola, J. Waddington, H. Walter, A. Waterreus, B. T. Webb, M. Weiser, N. M. Williams, S. H. Witt, B. K. Wormley, J. Q. Wu, Z. Xu, R. Yolken, C. C. Zai, W. Zhou, F. Zhu, F. Zimprich, E. C. Atbaşoğlu, M. Ayub, C. Benner, A. Bertolino, D. W. Black, N. J. Bray, G. Breen, N. G. Buccola, W. F. Byerley, W. J. Chen, C. R. Cloninger, B. Crespo-Facorro, G. Donohoe, R. Freedman, C. Galletly, M. J. Gandal, M. Gennarelli, D. M. Hougaard, H. G. Hwu, A. V. Jablensky, S. A. McCarroll, J. L. Moran, O. Mors, P. B. Mortensen, B. Müller-Myhsok, A. L. Neil, M. Nordentoft, M. T. Pato, T. L. Petryshen, M. Pirinen, A. E. Pulver, T. G. Schulze, J. M. Silverman, J. W. Smoller, E. A. Stahl, D. W. Tsuang, E. Vilella, S. H. Wang, S. Xu, R. Adolfsson, C. Arango, B. T. Baune, S. I. Belangero, A. D. Børglum, D. Braff, E. Bramon, J. D. Buxbaum, D. Campion, J. A. Cervilla, S. Cichon, D. A. Collier, A. Corvin, D. Curtis, M. D. Forti, E. Domenici, H. Ehrenreich, V. Escott-Price, T. Esko, A. H. Fanous, A. Gareeva, M. Gawlik, P. V. Gejman, M. Gill, S. J. Glatt, V. Golimbet, K. S. Hong, C. M. Hultman, S. E. Hyman, N. Iwata, E. G. Jönsson, R. S. Kahn, J. L. Kennedy, E. Khusnutdinova, G. Kirov, J. A. Knowles, M. O. Krebs, C. Laurent-Levinson, J. Lee, T. Lencz, D. F. Levinson, Q. S. Li, J. Liu, A. K. Malhotra, D. Malhotra, A. McIntosh, A. McQuillin, P. R. Menezes, V. A. Morgan, D. W. Morris, B. J. Mowry, R. M. Murray, V. Nimgaonkar, M. M. Nöthen, R. A. Ophoff, S. A. Paciga, A. Palotie, C. N. Pato, S. Qin, M. Rietschel, B. P. Riley, M. Rivera, D. Rujescu, M. C. Saka, A. R. Sanders, S. G. Schwab, A. Serretti, P. C. Sham, Y. Shi, D. St Clair, H. Stefánsson, K. Stefansson, M. T. Tsuang, J. van Os, M. P. Vawter, D. R. Weinberger, T. Werge, D. B. Wildenauer, X. Yu, W. Yue, P. A. Holmans, A. J. Pocklington, P. Roussos, E. Vassos, M. Verhage, P. M. Visscher, J. Yang, D. Posthuma, O. A. Andreassen, K. S. Kendler, M. J. Owen, N. R. Wray, M. J. Daly, H. Huang, B. M. Neale, P. F. Sullivan, S. Ripke, J. T. R. Walters, M. C. O'Donovan, *Nature* **2022**, *604* (7906), 502, https://doi.org/10.1038/s41586-022-04434-5.

[2] M. Fromer, P. Roussos, S. K. Sieberts, J. S. Johnson, D. H. Kavanagh, T. M. Perumal, D. M. Ruderfer, E. C. Oh, A. Topol, H. R. Shah, L. L. Klei, R. Kramer, D. Pinto, Z. H. Gümüş, A. E. Cicek, K. K. Dang, A. Browne, C. Lu, L. Xie, B. Readhead, E. A. Stahl, J. Xiao, M. Parvizi, T. Hamamsy, J. F. Fullard, Y. C. Wang, M. C. Mahajan, J. M. Derry, J. T. Dudley, S. E. Hemby, B. A. Logsdon, K. Talbot, T. Raj, D. A. Bennett, P. L. De Jager, J. Zhu, B. Zhang, P. F. Sullivan, A. Chess, S. M. Purcell, L. A. Shinobu, L. M. Mangravite, H. Toyoshiba, R. E. Gur, C. G. Hahn, D. A. Lewis, V. Haroutunian, M. A. Peters, B. K. Lipska, J. D. Buxbaum, E. E. Schadt, K. Hirai, K. Roeder, K. J. Brennand, N. Katsanis, E. Domenici, B. Devlin, P. Sklar, *Nat Neurosci* **2016**, *19* (11), 1442, https://doi.org/10.1038/nn.4399.

[3] A. E. Jaffe, R. E. Straub, J. H. Shin, R. Tao, Y. Gao, L. Collado-Torres, T. Kam-Thong, H. S. Xi, J. Quan, Q. Chen, C. Colantuoni, W. S. Ulrich, B. J. Maher, A. Deep-Soboslay, A. J. Cross, N. J. Brandon, J. T. Leek, T. M. Hyde, J. E. Kleinman, D. R. Weinberger, *Nat Neurosci* **2018**, *21* (8), 1117, https://doi.org/10.1038/s41593-018-0197-y.

[4] M. J. Gandal, P. Zhang, E. Hadjimichael, R. L. Walker, C. Chen, S. Liu, H. Won, H. van Bakel, M. Varghese, Y. Wang, A. W. Shieh, J. Haney, S. Parhami, J. Belmont, M. Kim, P. Moran Losada, Z. Khan, J. Mleczko, Y. Xia, R. Dai, D. Wang, Y. T. Yang, M. Xu, K. Fish, P. R. Hof, J. Warrell, D. Fitzgerald, K. White, A. E. Jaffe, M. A. Peters, M. Gerstein, C. Liu, L. M. Iakoucheva, D. Pinto, D. H. Geschwind, *Science* **2018**, *362* (6420), https://doi.org/10.1126/science.aat8127.

[5] C. Bycroft, C. Freeman, D. Petkova, G. Band, L. T. Elliott, K. Sharp, A. Motyer, D. Vukcevic, O. Delaneau, J. O'Connell, A. Cortes, S. Welsh, A. Young, M. Effingham, G. McVean, S. Leslie, N. Allen, P. Donnelly, J. Marchini, *Nature* **2018**, *562* (7726), 203, https://doi.org/10.1038/s41586-018-0579-z.

[6] S. E. Legge, H. J. Jones, K. M. Kendall, A. F. Pardiñas, G. Menzies, M. Bracher-Smith, V. Escott-Price, E. Rees, K. A. S. Davis, M. Hotopf, J. E. Savage, D. Posthuma, P. Holmans, G. Kirov, M. J. Owen, M. C. O'Donovan, S. Zammit, J. T. R. Walters, *JAMA Psychiatry* **2019**, *76* (12), 1256, https://doi.org/10.1001/jamapsychiatry.2019.2508.

[7] a) H. Yang, W. Chen, Y. Hu, Y. Chen, Y. Zeng, Y. Sun, Z. Ying, J. He, Y. Qu, D. Lu, F. Fang, U. A. Valdimarsdóttir, H. Song, *Lancet Healthy Longev* **2020**, *1* (2), e69, https://doi.org/10.1016/s2666-7568(20)30013-1; b) Y. Huang, D. Chen, A. M. Levin, B. K. Ahmedani, C. Frank, M. Li, Q. Wang, H. Gui, P. C. Sham, *Mol Psychiatry* **2023**, *28* (7), 2913, https://doi.org/10.1038/s41380-023-02124-w.

[8] a) A. Manichaikul, J. C. Mychaleckyj, S. S. Rich, K. Daly, M. Sale, W. M. Chen, *Bioinformatics* **2010**, *26* (22), 2867, https://doi.org/10.1093/bioinformatics/btq559; b) C. C. Chang, C. C. Chow, L. C. Tellier, S. Vattikuti, S. M. Purcell, J. J. Lee, *Gigascience* **2015**, *4*, 7, https://doi.org/10.1186/s13742-015-0047-8.

[9] I. Guyon, J. Weston, S. Barnhill, V. Vapnik, *Machine Learning* **2002**, *46* (1), 389, https://doi.org/10.1023/A:1012487302797.

[10] B. Boser, *ACM Press* **1992**.

[11] A. V.DavidSánchez, *Neurocomputing* **2003**, *55*, 5.

[12] D. Szklarczyk, A. L. Gable, D. Lyon, A. Junge, S. Wyder, J. Huerta-Cepas, M. Simonovic, N. T. Doncheva, J. H. Morris, P. Bork, L. J. Jensen, C. V. Mering, *Nucleic Acids Res* **2019**, *47* (D1), D607, https://doi.org/10.1093/nar/gky1131.

[13] R. Oughtred, C. Stark, B. J. Breitkreutz, J. Rust, L. Boucher, C. Chang, N. Kolas, L. O'Donnell, G. Leung, R. McAdam, F. Zhang, S. Dolma, A. Willems, J. Coulombe-Huntington, A. Chatr-Aryamontri, K. Dolinski, M. Tyers, *Nucleic Acids Res* **2019**, *47* (D1), D529, https://doi.org/10.1093/nar/gky1079.

[14] E. L. Huttlin, L. Ting, R. J. Bruckner, F. Gebreab, M. P. Gygi, J. Szpyt, S. Tam, G. Zarraga, G. Colby, K. Baltier, R. Dong, V. Guarani, L. P. Vaites, A. Ordureau, R. Rad, B. K. Erickson, M. Wühr, J. Chick, B. Zhai, D. Kolippakkam, J. Mintseris, R. A. Obar, T. Harris, S. Artavanis-Tsakonas, M. E. Sowa, P. De Camilli, J. A. Paulo, J. W. Harper, S. P. Gygi, *Cell* **2015**, *162* (2), 425, https://doi.org/10.1016/j.cell.2015.06.043.

[15] T. Rolland, M. Taşan, B. Charloteaux, S. J. Pevzner, Q. Zhong, N. Sahni, S. Yi, I. Lemmens, C. Fontanillo, R. Mosca, A. Kamburov, S. D. Ghiassian, X. Yang, L. Ghamsari, D. Balcha, B. E. Begg, P. Braun, M. Brehme, M. P. Broly, A. R. Carvunis, D. Convery-Zupan, R. Corominas, J. Coulombe-Huntington, E. Dann, M. Dreze, A. Dricot, C. Fan, E. Franzosa, F. Gebreab, B. J. Gutierrez, M. F. Hardy, M. Jin, S. Kang, R. Kiros, G. N. Lin, K. Luck, A. MacWilliams, J. Menche, R. R. Murray, A. Palagi, M. M. Poulin, X. Rambout, J. Rasla, P. Reichert, V. Romero, E. Ruyssinck, J. M. Sahalie, A. Scholz, A. A. Shah, A. Sharma, Y. Shen, K. Spirohn, S. Tam, A. O. Tejeda, S. A. Wanamaker, J. C. Twizere, K. Vega, J. Walsh, M. E. Cusick, Y. Xia, A. L. Barabási, L. M. Iakoucheva, P. Aloy, J. De Las Rivas, J. Tavernier, M. A. Calderwood, D. E. Hill, T. Hao, F. P. Roth, M. Vidal, *Cell* **2014**, *159* (5), 1212, https://doi.org/10.1016/j.cell.2014.10.050.

[16] J. Das, H. Yu, *BMC Syst Biol* **2012**, *6*, 92, https://doi.org/10.1186/1752-0509-6-92.

[17] T. S. Keshava Prasad, R. Goel, K. Kandasamy, S. Keerthikumar, S. Kumar, S. Mathivanan, D. Telikicherla, R. Raju, B. Shafreen, A. Venugopal, L. Balakrishnan, A. Marimuthu, S. Banerjee, D. S. Somanathan, A. Sebastian, S. Rani, S. Ray, C. J. Harrys Kishore, S. Kanth, M. Ahmed, M. K. Kashyap, R. Mohmood, Y. L. Ramachandra, V. Krishna, B. A. Rahiman, S. Mohan, P. Ranganathan, S. Ramabadran, R. Chaerkady, A. Pandey, *Nucleic Acids Res* **2009**, *37* (Database issue), D767, https://doi.org/10.1093/nar/gkn892.

[18] S. Orchard, M. Ammari, B. Aranda, L. Breuza, L. Briganti, F. Broackes-Carter, N. H. Campbell, G. Chavali, C. Chen, N. del-Toro, M. Duesbury, M. Dumousseau, E. Galeota, U. Hinz, M. Iannuccelli, S. Jagannathan, R. Jimenez, J. Khadake, A. Lagreid, L. Licata, R. C. Lovering, B. Meldal, A. N. Melidoni, M. Milagros, D. Peluso, L. Perfetto, P. Porras, A. Raghunath, S. Ricard-Blum, B. Roechert, A. Stutz, M. Tognolli, K. van Roey, G. Cesareni, H. Hermjakob, *Nucleic Acids Res* **2014**, *42* (Database issue), D358, https://doi.org/10.1093/nar/gkt1115.

[19] L. Licata, L. Briganti, D. Peluso, L. Perfetto, M. Iannuccelli, E. Galeota, F. Sacco, A. Palma, A. P. Nardozza, E. Santonico, L. Castagnoli, G. Cesareni, *Nucleic Acids Res* **2012**, *40* (Database issue), D857, https://doi.org/10.1093/nar/gkr930.

[20] J. Menche, A. Sharma, M. Kitsak, S. D. Ghiassian, M. Vidal, J. Loscalzo, A. L. Barabási, *Science* **2015**, *347* (6224), 1257601, https://doi.org/10.1126/science.1257601.

[21] P. Shannon, A. Markiel, O. Ozier, N. S. Baliga, J. T. Wang, D. Ramage, N. Amin, B. Schwikowski, T. Ideker, *Genome Res* **2003**, *13* (11), 2498, https://doi.org/10.1101/gr.1239303.

[22] T. Nepusz, H. Yu, A. Paccanaro, *Nature Methods* **2012**, *9* (5), 471, https://doi.org/10.1038/nmeth.1938.

[23] a) T. Onitsuka, M. E. Shenton, D. F. Salisbury, C. C. Dickey, K. Kasai, S. K. Toner, M. Frumin, R. Kikinis, F. A. Jolesz, R. W. McCarley, *Am J Psychiatry* **2004**, *161* (9), 1603, https://doi.org/10.1176/appi.ajp.161.9.1603; b) D. K. Shukla, S. A. Wijtenburg, H. Chen, J. J. Chiappelli, P. Kochunov, L. E. Hong, L. M. Rowland, *Schizophr Bull* **2019**, *45* (3), 647, https://doi.org/10.1093/schbul/sby075.

[24] J. Zhang, C. G. Abdallah, J. Wang, X. Wan, C. Liang, L. Jiang, Y. Liu, H. Huang, X. Hong, Q. Huang, R. Wu, C. Xu, *Psychiatry Res* **2012**, *200* (2-3), 126, https://doi.org/10.1016/j.psychres.2012.04.021.

[25] S. M. O'Donovan, C. Sullivan, R. Koene, E. Devine, K. Hasselfeld, C. L. Moody, R. E. McCullumsmith, *Neuropsychopharmacology* **2018**, *43* (8), 1667, https://doi.org/10.1038/s41386-018-0028-6.

[26] S. Mah, M. R. Nelson, L. E. Delisi, R. H. Reneland, N. Markward, M. R. James, D. R. Nyholt, N. Hayward, H. Handoko, B. Mowry, S. Kammerer, A. Braun, *Mol Psychiatry* **2006**, *11* (5), 471, https://doi.org/10.1038/sj.mp.4001785.

[27] L. K. Guo, Y. Su, Y. Y. Zhang, H. Yu, Z. Lu, W. Q. Li, Y. F. Yang, X. Xiao, H. Yan, T. L. Lu, J. Li, Y. D. Liao, Z. W. Kang, L. F. Wang, Y. Li, M. Li, B. Liu, H. L. Huang, L. X. Lv, Y. Yao, Y. L. Tan, G. Breen, I. Everall, H. X. Wang, Z. Huang, D. Zhang, W. H. Yue, *Mil Med Res* **2023**, *10* (1), 24, https://doi.org/10.1186/s40779-023-00459-7.

[28] C. S. Weickert, A. L. Miranda-Angulo, J. Wong, W. R. Perlman, S. E. Ward, V. Radhakrishna, R. E. Straub, D. R. Weinberger, J. E. Kleinman, *Hum Mol Genet* **2008**, *17* (15), 2293, https://doi.org/10.1093/hmg/ddn130.

[29] J. Sun, C. Wan, P. Jia, A. H. Fanous, K. S. Kendler, B. P. Riley, Z. Zhao, *Schizophr Res* **2011**, *125* (2-3), 201, https://doi.org/10.1016/j.schres.2010.12.002.

[30] M. Subbanna, V. Shivakumar, D. Venugopal, J. C. Narayanaswamy, M. Berk, S. Varambally, G. Venkatasubramanian, M. Debnath, *Psychiatry Clin Neurosci* **2020**, *74* (1), 64, https://doi.org/10.1111/pcn.12938.

[31] M. Kundakovic, *Trends Neurosci* **2022**, *45* (10), 716, https://doi.org/10.1016/j.tins.2022.06.001.

[32] D. Pérez-Rodríguez, M. A. Penedo, T. Rivera-Baltanás, T. Peña-Centeno, S. Burkhardt, A. Fischer, J. M. Prieto-González, J. M. Olivares, H. López-Fernández, R. C. Agís-Balboa, *Int J Mol Sci* **2023**, *24* (3), https://doi.org/10.3390/ijms24031891.

[33] Y. L. Liu, C. S. Fann, C. M. Liu, W. J. Chen, J. Y. Wu, S. I. Hung, C. H. Chen, Y. S. Jou, S. K. Liu, T. J. Hwang, M. H. Hsieh, C. C. Chang, W. C. Yang, J. J. Lin, F. H. Chou, S. V. Faraone, M. T. Tsuang, H. G. Hwu, *Biol Psychiatry* **2008**, *64* (9), 789, https://doi.org/10.1016/j.biopsych.2008.04.035.

[34] T. Ni, L. Zhu, S. Wang, W. Zhu, Y. Xue, Y. Zhu, D. Ma, H. Wang, F. Guan, T. Chen, *Mol Psychiatry* **2022**, *27* (10), 4009, https://doi.org/10.1038/s41380-022-01662-z.

[35] S. S. Rao, L. Lago, I. Volitakis, J. J. Shukla, G. McColl, D. I. Finkelstein, P. A. Adlard, *Neurotherapeutics* **2021**, *18* (2), 1081, https://doi.org/10.1007/s13311-020-00972-w.

[36] M. Chatterjee, M. Jaiswal, G. Palit, *ISRN Psychiatry* **2012**, *2012*, 595141, https://doi.org/10.5402/2012/595141.
